# Cerebral near-infrared spectroscopy monitoring (NIRS) in children and adults: a systematic review with meta-analysis

**DOI:** 10.1038/s41390-022-01995-z

**Published:** 2022-02-22

**Authors:** Mathias Lühr Hansen, Simon Hyttel-Sørensen, Janus Christian Jakobsen, Christian Gluud, Elisabeth M. W. Kooi, Jonathan Mintzer, Willem P. de Boode, Monica Fumagalli, Ana Alarcon, Thomas Alderliesten, Gorm Greisen, on behalf of the European Society for Paediatric Research Special Interest Group ‘NearInfraRed Spectroscopy’ (NIRS), Mathias Lühr Hansen, Elisabeth M. W. Kooi, Jonathan Mintzer, Monica Fumagalli, Ana Alarcon, Thomas Alderliesten, Gorm Greisen, Topun Austin, Marlies Bruckner, Willem P. de Boode, Eugene Dempsey, Ebru Ergenekon, Kivilcim Gucuyener, Philip T. Levy, Kian D. Liem, Silvia Martini, Gunnar Naulaers, Felix Neunhoeffer, Adelina Pellicer, Gerhard Pichler, Charles Christoph Roehr, Claudia Roll, Christoph E. Schwarz, Tomasz Szczapa, Berndt Urlesberger, Martin Wolf, Flora Wong, Christopher J. Rhee, Petra Lemmers

**Affiliations:** 1 Department of Neonatology, Copenhagen University Hospital – Rigshospitalet, Blegdamsvej 9, 2100 Copenhagen, Denmark; 2 Department of Intensive Care, Copenhagen University Hospital – Rigshospitalet, Blegdamsvej 9, 2100 Copenhagen, Denmark; 3 Copenhagen Trial Unit, Centre for Clinical Intervention Research, The Capital Region, Copenhagen University Hospital – Rigshospitalet, Blegdamsvej 9, 2100 Copenhagen, Denmark; 4 Department of Regional Health Research, The Faculty of Health Sciences, University of Southern Denmark, Odense, Denmark; 5 Division of Neonatology, University of Groningen, University Medical Center Groningen, Beatrix Children’s Hospital, Groningen, the Netherlands; 6 Department of Pediatrics, Division of Newborn Medicine, Mountainside Medical Center, Montclair, NJ USA; 7 Division of Neonatology, Department of Pediatrics, Radboud University Medical Center, Radboud Institute for Health Sciences, Amalia Children’s Hospital, Nijmegen, the Netherlands; 8 Fondazione IRCCS Ca’ Granda Ospedale Maggiore Policlinico Milan, Via Francesco Sforza 35, 20122 Milano, Italy; 9 Department of Clinical Sciences and Community Health, University of Milan, Via Festa del Perdono 7, 20122 Milano, Italy; 10 Department of Neonatology, Hospital Sant Joan de Deu, Passeig de Sant Joan de Deu 2, 08950 Esplugues de Llobregat, Barcelona Spain; 11 Department of Neonatology, University Medical Center Utrecht Brain Center, Utrecht University, Utrecht, the Netherlands; 12 Topun Austin, Neonatal Intensive Care Unit, Cambridge University Hospitals NHS Foundation Trust, Cambridge, UK; 13 Research Unit for Neonatal Micro- and Macrocirculation, Department of Pediatrics and Adolescent Medicine, Medical University of Graz, Graz, Austria; 14 Department of Paediatrics and Child Health, INFANT Centre, University College Cork, Cork, Ireland; 15 Division of Newborn Medicine, Department of Pediatrics, Gazi University Hospital, Ankara, Turkey; 16 Department of Pediatric Neurology, Gazi University Hospital, Ankara, Turkey; 17 Boston Children’s Hospital, Harvard Medical School, Harvard University, Boston, MA USA; 18 Department of Neonatology, Radboud University Medical Center, Radboud Institute for Health Sciences, Amalia Children’s Hospital, Nijmegen, the Netherlands; 19 Department of Medical and Surgical Sciences, Neonatal Intensive Care Unit, S. Orsola-Malpighi University Hospital, University of Bologna, Bologna, Italy; 20 Department of Development and Regeneration, Woman and Child, KU Leuven, Leuven, Belgium; 21 Department of Pediatric Cardiology, Pulmonology and Pediatric Intensive Care Medicine, University Children’s Hospital Tübingen, Tübingen, Germany; 22 Department of Neonatology, La Paz University Hospital, Madrid, Spain; 23 Department of Pediatrics, Medical University of Graz, Graz, Austria; 24 Newborn Services, Southmead Hospital, North Bristol Trust, Bristol, UK; 25 National Perinatal Epidemiology Unit Clinical Trials Unit, Nuffield Department of Population Health, Medical Sciences Division, University of Oxford, Oxford, UK; 26 Faculty of Health Sciences, University of Bristol, Bristol, UK; 27 Department of Neonatology, Pediatric Intensive Care, Sleep Medicine, Vest Children’s Hospital Datteln, University Witten-Herdecke, Datteln, Germany; 28 Department of Neonatology, University Children’s Hospital Tübingen, Tübingen, Germany; 29 Department of Neonatology, Biophysical Monitoring and Cardiopulmonary Therapies Research Unit, Poznan University of Medical Sciences, Poznan, Poland; 30 Division of Neonatology, Department of Pediatrics and Adolescent Medicine, Medical University of Graz, Graz, Austria; 31 Biomedical Optics Research Laboratory, Department of Neonatology, University Hospital Zurich, Zurich, Switzerland; 32 Monash Newborn, Monash Children’s Hospital, Hudson Institute of Medical Research, Department of Paediatrics, Monash University, Melbourne, VIC Australia; 33 Section of Neonatology, Baylor College of Medicine, Texas Children’s Hospital, Houston, TX USA; 34 Department of Neonatology, Wilhelmina Children’s Hospital, University Medical Center, Utrecht, the Netherlands

## Abstract

**Background:**

Cerebral oxygenation monitoring utilising near-infrared spectroscopy (NIRS) is increasingly used to guide interventions in clinical care. The objective of this systematic review with meta-analysis and Trial Sequential Analysis is to evaluate the effects of clinical care with access to cerebral NIRS monitoring in children and adults versus care without.

**Methods:**

This review conforms to PRISMA guidelines and was registered in PROSPERO (CRD42020202986). Methods are outlined in our protocol (doi: 10.1186/s13643-021-01660-2).

**Results:**

Twenty-five randomised clinical trials were included (2606 participants). All trials were at a high risk of bias. Two trials assessed the effects of NIRS during neonatal intensive care, 13 during cardiac surgery, 9 during non-cardiac surgery and 1 during neurocritical care. Meta-analyses showed no significant difference for all-cause mortality (RR 0.75, 95% CI 0.51–1.10; 1489 participants; *I*^2^ = 0; 11 trials; very low certainty of evidence); moderate or severe, persistent cognitive or neurological deficit (RR 0.74, 95% CI 0.42–1.32; 1135 participants; *I*^2^ = 39.6; 9 trials; very low certainty of evidence); and serious adverse events (RR 0.82; 95% CI 0.67–1.01; 2132 participants; *I*^2^ = 68.4; 17 trials; very low certainty of evidence).

**Conclusion:**

The evidence on the effects of clinical care with access to cerebral NIRS monitoring is very uncertain.

**Impact:**

- The evidence of the effects of cerebral NIRS versus no NIRS monitoring are very uncertain for mortality, neuroprotection, and serious adverse events. Additional trials to obtain sufficient information size, focusing on lowering bias risk, are required.
- The first attempt to systematically review randomised clinical trials with meta-analysis to evaluate the effects of cerebral NIRS monitoring by pooling data across various clinical settings.
- Despite pooling data across clinical settings, study interpretation was not substantially impacted by heterogeneity.
- We have insufficient evidence to support or reject the clinical use of cerebral NIRS monitoring.

## Introduction

The primary purpose of cerebral oxygenation monitoring by near-infrared spectroscopy (NIRS) is to allow for timely interventions to prevent cerebral hypoxia and subsequent brain injury,^1–3^ which, in severe cases, can lead to death.^4,5^ Despite not being part of standard care on a broad scale, the use of cerebral NIRS monitoring is increasing across various clinical settings.^3,6–11^ In neonatal intensive care, a survey from 2015 demonstrated that 69/235 neonatal intensive care units from Australia, Asia, and North America used cerebral NIRS monitoring in the clinical setting.^7^ In addition, neonatal intensive care units from the United States, Brazil, and Korea have reported to routinely use cerebral NIRS monitoring for specific indications.^6,10^ In paediatric intensive care, it has been reported that cerebral NIRS monitoring is part of standard care within several institutions in the United States.^11^ In adult intensive care, cerebral NIRS monitoring is mostly limited to research purposes.^11^ As a perioperative monitoring tool during cardiac surgery, cerebral NIRS monitoring has been recommended based on a Delphi consensus statement, by the American Society for Enhanced Recovery and the Perioperative Quality Initiative^12^ and is widely used across all age groups.^3,8,9,13^ For non-cardiac surgery, the use of cerebral NIRS monitoring is clinical care limited and cannot currently be considered a standard practice.^3,12^ Previous systematic reviews with meta-analysis have assessed the effects of clinical care with access to cerebral NIRS monitoring in specific clinical settings, including neonatal intensive care in very preterm infants,^14^ cardiopulmonary bypass surgery in adults,^15^ and all types of surgery in children and adults.^16^ All three reviews conclude that the existing evidence does not show a benefit of cerebral NIRS monitoring. This is primarily due to a lack of published trials at low risk of bias, but also due to the low number of clinically relevant events.^14–16^ As the occurrence of brain injury caused by cerebral hypoxia and mortality typically is low, especially during surgery,^15,16^ it is difficult to reach a sufficient information size within the individual clinical settings.^17,18^ Classifying and pooling neurological outcomes based on severity, along with pooling mortality, from randomised trials across various clinical settings might enable us to reach a sufficient number of events and thus, a sufficient information size.^19^ However, pooling randomised trials from different clinical settings is also problematic, as substantial clinical heterogeneity can be expected, and interpretation of such analyses for specific clinical settings might be difficult. Thus, a beneficial effect from such analyses would mainly serve as a ‘signal’ of benefit from cerebral NIRS monitoring, encouraging the planning and conduct of future randomised clinical trials within the specific clinical settings, until sufficient information sizes will be reached.^19^

The objective of this systematic review with meta-analysis and Trial Sequential Analysis (TSA), was to evaluate the effects of clinical care with access to cerebral NIRS monitoring versus clinical care without access to cerebral NIRS monitoring in children and adults across all clinical settings.^19^

## Methods

The reporting of this systematic review is in adherence with the Preferred Reporting Items for Systematic Reviews and Meta-Analysis guidelines (PRISMA)^20^ (see PRISMA checklist in Appendix A) and was registered in PROSPERO (CRD42020202986). The predefined methodology is based on the Cochrane Handbook for Systematic Reviews of Interventional Research,^21^ and is described in detail in our published protocol.^19^

### Eligibility criteria

We searched for and included randomised clinical trials evaluating the effect of clinical care with access to cerebral NIRS monitoring versus clinical care without access to cerebral NIRS monitoring in children and adults across all clinical settings.^19^ Only trials investigating cerebral oximetry in combination with a treatment guideline, targeting cerebral oxygenation, are included. This excludes:

1. Trials that are testing a ‘monitoring package’, e.g., the combination of intraoperative BiSpectral index and cerebral oximetry monitoring to guide treatment.^22^
2. An additional experimental treatment element, besides cerebral oximetry, in the experimental group, e.g., specific intervention thresholds for routinely used monitoring parameters that are only implemented in the experimental group.
3. An experimental intervention in the control group, e.g., trials that do not compare the cerebral oximetry intervention with usual care or ‘placebo’.

### Outcome definitions

Our three primary outcomes were all-cause mortality at maximal follow-up; moderate or severe, persistent cognitive or neurological deficit, significantly affecting daily life, at maximal follow-up (e.g., stroke; Bayley Scale of Infant Development score below minus two standard deviation at 2 years or later^23^) and proportion of participants with one or more serious adverse events.^24^ Secondary outcomes were mild, moderate or severe, temporary or persistent, cognitive or neurological deficit (e.g., postoperative delirium, abnormal general movements at term age); quality of life at maximal follow-up; brain damage on imaging at maximal follow-up; and adverse events.^24^ Exploratory outcomes were any evidence of a negative impact on the brain; individual serious and non-serious adverse events.^19,24^ A detailed description can be found in our protocol.^19^

### Outcome classification and pooling

Two authors MLH and SH-S identified the relevant outcome measures from the included trials, and presented them to the authors GG and CG, who, blinded to the data, then classified and pooled them according to the outcome definitions as stated above, and as described in detail in the protocol.^19^ In cases of disagreement between GG and CG, JCJ made the final decision.

### Search strategy, study selection and data extraction

A two-step search strategy was used. First, a ‘combined search’ was conducted to identify eligible randomised clinical trials in a simple, effective manner. The combined search included (1) searching the reference lists of previously published systematic reviews;^15,16,25^ (2) searching clinicaltrials.gov as outlined in the protocol^19^ (November 2020); and (3) searching PubMed as outlined in ‘Appendix B: Search strategy’ (November 2020).

To ensure that no eligible trials were missed, we also conducted a systematic search in MEDLINE, to identify eligible randomised clinical trials. The search in MEDLINE was conducted from inception and onwards up until 30 March 2021, and as described in the protocol and in ‘Appendix B: Search strategy’.^19^ We also checked the reference lists of the relevant publications, to identify additional relevant trials. Trials were included, regardless of trial design, publication status, year of publication, language of publication, and outcome reporting.^19^

The literature search and study selection were conducted by MLH who, if in doubt regarding the eligibility of studies, consulted with GG or JCJ. Data extraction was conducted independently by the authors MLH and SH-S, based on a predefined data extraction form. If relevant data were missing, or if the included trials did not report all the prespecified outcomes, the trialists were contacted. The published protocol also includes a detailed description of the data collection process.^19^ The extracted data are available in ‘Appendix C: Characteristics of trials, data extraction and risk of bias assessment’.

### Assessment of risk of bias

Based on the Cochrane risk of bias tool – version 2,^26^ MLH and SH-S conducted independent risk of bias assessment of all included trials and for each outcome within the trials. The domains were bias from the randomisation process, bias due to deviation from intended interventions, bias due to missing outcome data, bias in the measurement of outcomes, and bias in the selection of the reported results.^21,26^ All trials reporting outcomes classified as primary outcomes in this systematic review were assessed for publication, and ‘for-profit’ (industry funding), bias as well.^27^

### Data synthesis

Meta-analyses were conducted as recommended in the Cochrane Handbook for Systematic Reviews of Interventions.^21^ To assess if the boundaries for statistical as well as clinical significance were crossed, the eight-step procedure by Jakobsen et al. was used.^18^ All statistical analyses were performed in Stata 17 (StataCorp LLC, College Station, Texas). Risk ratios were calculated for dichotomous outcomes and standardised mean difference was intended to be calculated for the single continuous outcome. For the primary analysis of all outcomes, fixed-effect (Mantel–Haenszel model)^28^ and random-effects (DerSimonian Laird model)^29^ meta-analyses were conducted, and the most conservative result was reported as the primary result (and primary analysis model). The three primary outcomes also underwent TSA.^17^ If the trial sequential boundaries for futility, benefit or harm were not crossed, or if the required information size was not reached, the TSA-adjusted confidence intervals (CI) were reported.^17,30^ Since we report on three primary outcomes, a *p* value of 0.025 was chosen as the threshold for statistical significance for each of the primary outcomes.^18^ A *p* value of 0.05 was chosen as the threshold for the hypothesis-generating secondary and exploratory outcomes. For the TSA, an alpha of 2.5%, a beta of 10%, and a relative risk reduction of 20% as the anticipated intervention effect were used for all three primary outcomes. The Bayes factor^31^ was calculated for three primary outcomes and a value of 0.1, at an anticipated risk reduction of 20%, was chosen as the threshold for determining if the meta-analyses results were most compatible with the null- or the alternative hypothesis.^18^ To determine the potential impact of missing data, we conducted ‘best-worst’ and ‘worst-best’ case scenario analyses for the three primary outcomes.^18^ The Grading of Recommendations Assessment, Development and Evaluation (GRADE) approach was used to assess the quality of the body of evidence for the primary outcomes, including the risk of bias assessments,^32^ heterogeneity or inconsistency of results,^33^ imprecision,^34^ indirectness,^35^ and publication bias.^27^

### Subgroup analyses

As outlined in the protocol, the following subgroup analyses were pre-planned and conducted when possible: risk of bias (high versus low); clinical settings (e.g., neonatal intensive care, cardiac surgery, non-cardiac surgery); industry support (no industry funding versus industry funding); and cerebral NIRS monitoring in the control group (blinded versus no blinding).^19^ The meta-analysis model used in the primary analysis for each primary outcome was also used for the subgroup analyses.

## Results

### Included trials

**Fig. 1:**
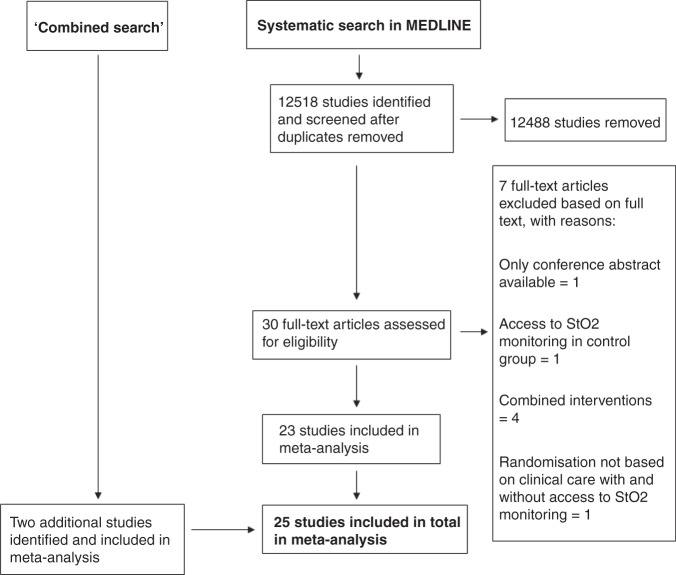
PRISMA flowchart. PRISMA: Preferred Reporting Items for Systematic Reviews and Meta-Analyses, StO2: tissue oxygen saturation.

The systematic search in MEDLINE identified 12,518 studies after the duplicate screening. Based on the available abstract or title, 12,488 studies were excluded. Of the remaining 30 studies, seven were excluded due to only conference abstracts being available,^36^ access to cerebral NIRS monitoring for participants in the control group if prolonged cerebral hypoxia occurred,^37^ clinical care with access to cerebral NIRS monitoring combined with an additional intervention,^22,38–40^ randomisation based on sensor position, and no clinical care based on the cerebral NIRS monitoring.^41^ Thus, a total of 23 trials in the systematic MEDLINE search matched our eligibility criteria and were included.^42–64^ The ‘combined search’ identified two additional trials not identified in the systematic search and matching our inclusion criteria.^65,66^ Therefore, a total of 25 trials randomising a total of 2606 participants were included.^42–66^ For characteristics of included and excluded studies, see ‘Appendix C: Characteristics of trials, data extraction and risk of bias assessment’. All trials were written in English, except for one, which was written in Chinese.^63^ An overview of the literature search can be found in the PRISMA flowchart (Fig. 1). All 25 trials were overall at high risk of bias. Regarding the risk of bias on individual outcomes, brain injury on cranial ultrasound in Hyttel-Sørensen et al.^60^ and serious permanent stroke in Rogers et al.^64^ were considered to be at low risk of bias. All other outcomes were at high risk of bias. An overview of the risk of bias assessment is provided in ‘Appendix C: Characteristics of trials, data extraction, and risk of bias assessment’.

#### Cardiac surgery

Thirteen of the included trials assessed the effects of clinical care with access to intraoperative cerebral NIRS monitoring in adults during cardiac surgery.^42,44–49,51,52,64,65^ In these trials, interventions were considered if cerebral oxygenation dropped below a predefined number of percentage points from baseline (10–30% drop). In three trials, interventions were also considered if cerebral oxygenation dropped below an absolute value of 50%.^42,50,64^ In one trial, cerebral NIRS monitoring was continued postoperatively during the first 24 h in the intensive care unit to guide interventions.^47^ In one of the cardiac surgery trials, cerebral NIRS monitoring was used to guide blood transfusions.^52^

#### Non-cardiac surgery

Nine of the included trials assessed the effects of clinical care with access to intraoperative cerebral NIRS monitoring in adults during non-cardiac surgery (orthopaedic, abdominal, aortic arch, spinal and carotid surgery).^53–59,63,66^ As in the cardiac surgery trials, interventions were considered if cerebral oxygenation dropped below a predefined number of percentage points from baseline (10–25% drop). In three trials, interventions should also be considered if the cerebral oxygenation dropped below an absolute value of 50 or 55%.^56,57,66^ One trial included two experimental NIRS arms; in one arm, interventions were based on a strict and predefined treatment algorithm, while in the other arm, no formal treatment algorithm to guide interventions was provided.^59^ In one trial, interventions were aimed at maintaining cerebral oxygenation of 63% (±3%) or cerebral oxygenation no less than 10% from baseline while controlling blood pressure in hypertensive elderly patients undergoing spinal surgery.^63^

#### Neonatal intensive care

Two of the included trials assessed the effects of clinical care with access to cerebral NIRS monitoring during neonatal intensive care, i.e., cardiopulmonary support after birth and in the neonatal period. In one trial, extremely preterm infants (born <28 weeks of gestational age) underwent cerebral NIRS monitoring for the first 72 h of life in the neonatal intensive care unit to guide interventions when the cerebral oxygenation dropped below 55% or increased above 85%.^60^ In the second trial, preterm infants (born <34 weeks of gestational age) underwent cerebral NIRS monitoring in the delivery room for the first 15 min after birth. Interventions were initiated when the cerebral oxygenation dropped below a predefined 10th percentile threshold or increased above a 90th percentile threshold.^61^

#### Neurocritical care

One of the included trials assessed the effects of clinical care with access to cerebral NIRS monitoring in neurocritical patients with traumatic brain injury, subarachnoid haemorrhage or intracerebral haemorrhage.^62^ In this trial, cerebral NIRS monitoring was used to guide blood transfusions.

For detailed characteristics of the included trials, see ‘Appendix C: Characteristics of trials, data extraction, and risk of bias assessment’. GRADE and the diversity-adjusted required information sizes (DARIS) are illustrated in Table 1 for the three primary outcomes.

**Table 1:** Summary of findings table.

Summary of findings:						
Clinical care with access to cerebral NIRS monitoring compared to clinical care without access to cerebral NIRS monitoring in children and adults						
Patient or population: children and adults Setting: hospital Intervention: clinical care with access to cerebral NIRS monitoring Comparison: clinical care without access to cerebral NIRS monitoring						
Outcomes	Anticipated absolute effects^a^ (95% CI)		Relative effect (95% CI)	No. of participants (studies)	Certainty of the evidence (GRADE)	Comments
	Risk with clinical care without access to cerebral NIRS monitoring	Risk with clinical care with access to cerebral NIRS monitoring				
All-cause mortality maximum follow-up (all-cause mortality)	72 per 1000	54 per 1000 (37–79)	RR 0.75 (0.51–1.10)	1489 (11 RCTs)	⨁◯◯◯ Very low	Downgraded one level due to serious risk of bias and two levels for very serious imprecision due to the small information size (DARIS 14,509: Pc 7.2%; RR 20%; alpha 2.5%; beta 10%; diversity 0%)
Moderate or severe, persistent cognitive or neurological deficit, significantly affecting daily life at maximum follow-up (moderate or severe, persistent cognitive or neurologic deficit)	138 per 1000	102 per 1000 (58–182)	RR 0.74 (0.42–1.32)	1135 (9 RCTs)	⨁◯◯◯ Very low	Downgraded one level due to serious risk of bias and two levels for very serious imprecision due to the small information size (DARIS 33,656: Pc 13.8%; RR 20%; alpha 2.5%; beta 10%; diversity 78.9%)
Proportion of participants with one or more serious adverse events (serious adverse events)	424 per 1000	348 per 1000 (284–429)	RR 0.82 (0.67–1.01)	2132 (17 RCTs)	⨁◯◯◯ Very low	Downgraded one level due to serious risk of bias, one level for serious inconsistency due to significant heterogeneity despite excluding Mohandas et al. (2013) that had the largest effect size, and substantial variability in the effect estimates, and one level for serious imprecision due to the small information size (DARIS 7685: Pc 42.4%; RR 20%; alpha 2.5%; beta 10%; diversity 78.8%)

GRADE Working Group grades of evidence: High certainty: we are very confident that the true effect lies close to that of the estimate of the effect. Moderate certainty: we are moderately confident in the effect estimate: the true effect is likely to be close to the estimate of the effect, but there is a possibility that it is substantially different. Low certainty: our confidence in the effect estimate is limited: the true effect may be substantially different from the estimate of the effect. Very low certainty: we have very little confidence in the effect estimate: the true effect is likely to be substantially different from the estimate of effect.

*CI* confidence interval, *RR* risk ratio.

^a^The risk in the intervention group (and its 95% confidence interval) is based on the assumed risk in the comparison group and the relative effect of the intervention (and its 95% CI).

### Effects of interventions

#### Primary outcomes

##### All-cause mortality

**Fig. 2:**
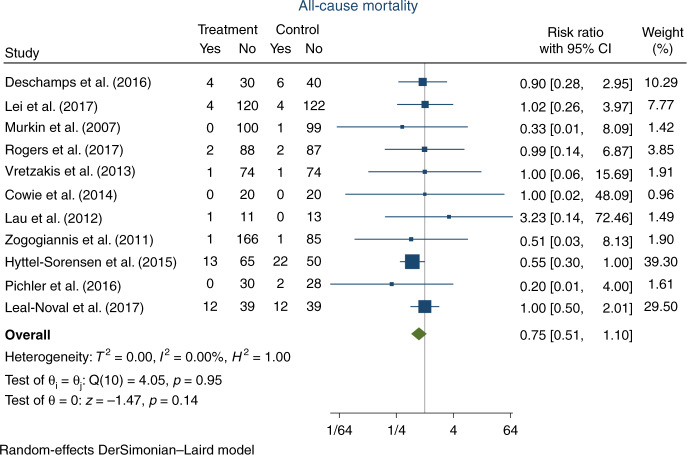
Forest plot of all-cause mortality. CI confidence interval.

**Fig. 3:**
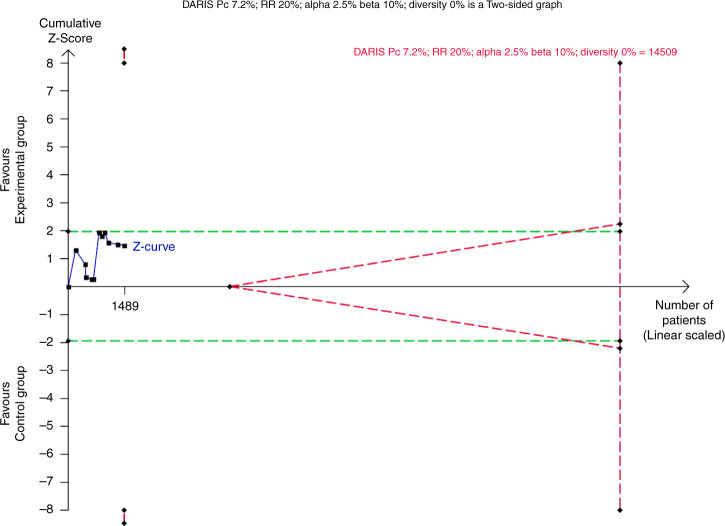
Trial Sequential Analysis of all-cause mortality. The Trial Sequential Analysis of all-cause mortality showed that the required information size to confirm or reject that adding cerebral NIRS monitoring reduced the relative risk of death by 20% was 14,509 participants, using a 7.2% mortality in the control group, an alpha of 2.5%, a beta of 10%, and the diversity of 0% found in the meta-analysis. The accrued information size was 1489 participants, which was compatible with a reduced risk of death by 84% or an increased risk of death by 263% (TSA-adjusted CI 0.16–3.63). Thus, the trial sequential monitoring boundaries for benefit, harm or futility were not crossed. DARIS diversity-adjusted required information size, Pc proportion in control group, RR relative risk.

Eleven trials, randomising a total of 1534 participants, reported on all-cause mortality (45 participants comprising 20 in the experimental group versus 25 in the control group were considered lost to follow-up due to no reported data on mortality status). In the experimental group, 38/781 (4.9%) participants died, versus 51/708 (7.2%) in the control group. Meta-analysis showed no significant difference in all-cause mortality (RR 0.75, 95% CI 0.51–1.10; *p* = 0.1; *I*^2^ = 0%; 1489 participants; 11 trials; Fig. 2; Bayes factor 0.37). The TSA showed that inadequate information existed to confirm or reject that the intervention reduced the risk of death by 20% (10.3% of the required information size had been accrued, TSA-adjusted CI 0.16–3.63, Fig. 3). The ‘best-worst case’ and ‘worst-best case’ scenarios showed that missing data alone had the potential to bias the results (Appendix D, Supplementary Figs. S7 and S8). This outcome result was assessed as a high risk of bias and the certainty of the evidence was considered as very low due to the high risk of bias and very serious imprecision (Table 1). None of the pre-planned subgroup analyses showed a significant difference in intervention effects between the subgroups (Appendix D, Supplementary Figs. S3–S5). Visual inspection of the funnel plot showed no signs of publication bias (Appendix D, Supplementary Fig. S9).

##### Moderate or severe, persistent cognitive or neurological deficit, significantly affecting daily life, at maximum follow-up

**Fig. 4:**
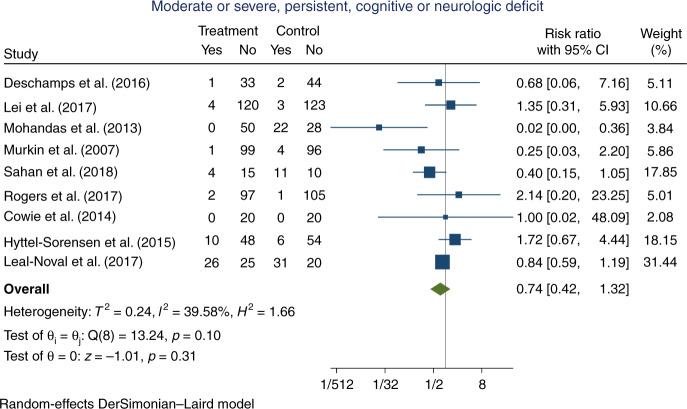
Forest plot of moderate-to-severe, persistent cognitive or neurological deficit, significantly affecting daily life, at maximum follow-up. CI confidence interval.

Nine trials, randomising a total of 1192 participants, reported an outcome that was classified as ‘moderate or severe, persistent cognitive or neurological deficit, significantly affecting daily life, at maximum follow-up’ (57 participants comprising 33 in the experimental group and 24 in the control group were considered lost to follow-up due to no reporting on the outcome data). The classified outcomes included stroke up until 5 or 30 days postoperative,^44,49,55^ stroke with uncertain exact assessment time,^47,64^ postoperative cognitive decline at 3 months postoperative,^48,50^ survival with moderate-to-severe neurodevelopmental impairment at 2 years of age,^60^ or a Glasgow Outcome Score of 2 or 3 at discharge.^62^ In the experimental group, 48/555 (8.6%) participants suffered an event versus 80/580 (13.8%) in the control group. Meta-analysis showed no significant difference on the outcome (RR 0.74, 95% CI 0.42–1.32; *p* = 0.31; *I*^2^ = 39.6%; 1135 participants; 9 trials; Fig. 4; Bayes factor 0.60). Although the statistical heterogeneity was not statistically significant (*I*^2^ = 39.6%; *p* = 0.10), visual inspection of the forest plot suggested that Mohandas et al.^48^ was an outlier, and a sensitivity analysis excluding this trial reduced *I*^2^ to 0% (RR 0.85, 95% CI 0.64–1.14; *p* = 0.27) (Appendix D, Supplementary Fig. S19). The clinical characteristics of Mohandas et al. did not differ substantially from the additional trials (Appendix C: Characteristics of trials, data extraction, and risk of bias assessment). The TSA showed that inadequate information existed to confirm or reject that the intervention reduced the risk of moderate or severe, persistent, cognitive or neurological deficit by 20% (3.4% of the required information size had been accrued, TSA-adjusted CIs could not be determined due to the small information size, Appendix D, Supplementary Fig. S16). The ‘best-worst case’ and ‘worst-best case’ scenarios showed that missing data alone had the potential to bias the results (Appendix D, Supplementary Figs. S17 and S18). This outcome result was assessed as high risk of bias and the certainty of the evidence was considered as very low due to the high risk of bias and very serious imprecision (Table 1). None of the pre-planned subgroup analyses showed a significant difference in intervention effects between the subgroups (Appendix D, Supplementary Figs. S12–S15). Since less than ten trials were included in the meta-analysis for this outcome, no assessment of publication bias was conducted.^19^

##### Proportion of participants with one or more serious adverse events

**Fig. 5:**
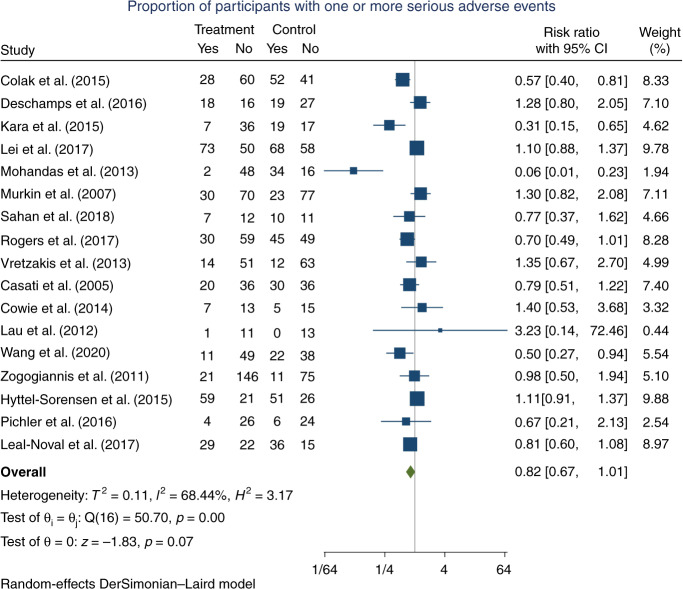
Forest plot of proportion of participants with one or more serious adverse events. CI confidence interval.

**Fig. 6:**
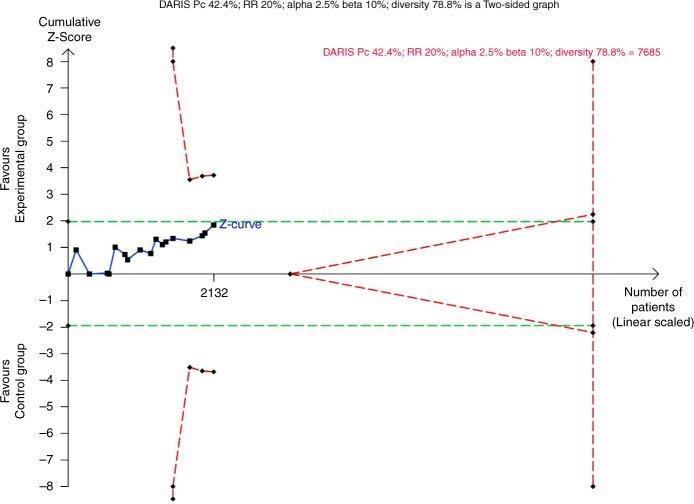
Trial Sequential Analysis of proportion of participants with one or more serious adverse events. The Trial Sequential Analysis of serious adverse events showed that the required information size to confirm or reject that adding cerebral NIRS monitoring reduced the relative risk of suffering one or more serious adverse events by 20% was 7685 participants, using a 42.4% event proportion in the control group, an alpha of 2.5%, a beta of 10%, and the diversity of 78.8% found in the meta-analysis. The accrued information size was 2132 participants, which was compatible with a reduced risk of suffering one or more serious adverse events by 44% or an increased risk of suffering one or more serious adverse events by 20% (TSA-adjusted CI 0.56–1.20). Thus, the trial sequential monitoring boundaries for benefit, harm or futility were not crossed. DARIS diversity-adjusted required information size, Pc proportion in control group, RR relative risk.

Seventeen trials, randomising a total of 2200 participants, reported one or more outcomes, classified as a serious adverse event (68 participants comprising 40 in the experimental group and 28 in the control group were considered lost to follow-up due to no reporting on outcomes classified as serious adverse events). For an overview of the events reported in each trial, see ‘Appendix C: Characteristics of trials, data extraction and risk of bias assessment’. In the experimental group, 361/1088 (33.2%) participants had one or more serious adverse events versus 443/1044 (42.4%) in the control group. Meta-analysis showed no significant difference on the outcome (RR 0.82, 95% CI 0.67–1.01; *p* = 0.07; *I*^2^ = 68.4%; 2132 participants; 17 trials; Fig. 5); Bayes factor 0.14. Testing for statistical heterogeneity was significant (*I*^2^ = 68.4%, *p* = 0.00). Based on visual inspection of the forest plot, the results from Mohandas et al.^48^ was suspected for being the main reason for heterogeneity. After excluding Mohandas et al. in a sensitivity analysis, heterogeneity was still statistically significant (*I*^2^ = 57.4%, *p* = 0.00) (Appendix D, Supplementary Fig. S29). The TSA showed that inadequate information existed to confirm or reject that the intervention reduced the risk of one or more serious adverse events, by 20% (27.7% of the required information size had been accrued, TSA-adjusted CI 0.56–1.20, Fig. 6). The ‘best-worst case’ and ‘worst-best case’ scenarios showed that missing data alone had the potential to bias the results (Appendix D, Supplementary Figs. S27 and S28). This outcome result was assessed as a high risk of bias and the certainty of the evidence was considered as very low due to the high risk of bias, serious inconsistency, and serious imprecision (Table 1). Publication bias was also suspected, as the funnel plot revealed asymmetry (Appendix D, Supplementary Fig. S30). However, the Harbord test^67^ was insignificant (*p* = 0.6). A significant interaction was observed for the subgroup analyses on the risk of bias and industry funding. The additional pre-planned subgroup analyses showed no significant group differences (Appendix D, Supplementary Figs. S22–S25).

#### Secondary outcomes

##### Mild, moderate or severe, temporary or persistent, cognitive or neurological deficit

Seventeen trials randomising a total of 2134 participants, reported an outcome that was classified as mild, moderate or severe, temporary or persistent, cognitive or neurological deficit (115 participants comprising 67 in the experimental group and 48 in the control group were considered lost to follow-up due to no reporting of outcome data). The classified outcomes included postoperative cognitive impairment 5–7 days postoperative,^42,65^ postoperative cognitive impairment at uncertain assessment time,^46^ postoperative cognitive decline 2–7 days postoperative^48,54,57^ postoperative cognitive dysfunction seven days postoperative,^50^ postoperative delirium 7–30 days postoperative or discharge^44,47,63^ short-term postoperative neurologic deficit (assessment time is uncertain),^59^ stroke 5–30 days postoperative,^49,55^ permanent stroke with uncertain assessment time,^64^ moderate-to-severe neurodevelopmental impairment at 2 years of age,^60^ abnormal general movements at discharge or term age,^61^ or unfavourable Glasgow outcome scale score 2 or 3 at discharge.^62^ In the experimental group, 182/1044 (17.4%) suffered an event versus 278/975 (28.5%) in the control group. Meta-analysis showed a significant difference on the outcome (RR 0.66, 95% CI 0.51–0.84; *p* = 0.00; *I*^2^ = 45.5%; 2019 participants; 17 trials; Appendix D, Supplementary Fig. S32). This outcome result was considered at high risk of bias (Appendix C: Characteristics of trials, data extraction and risk of bias assessments). Testing for statistical heterogeneity was significant (*I*^2^ = 45.5%, *p* = 0.02). Based on visual inspection of the forest plot, the results from Mohandas et al.^48^ was suspected as being the main reason for heterogeneity. After excluding Mohandas et al. in a sensitivity analysis, heterogeneity was insignificant (*I*^2^ = 10.2%, *p* = 0.34), and the intervention effect estimate was consistent with the primary analysis (RR 0.72, 95% CI 0.60–0.85) (Appendix D, Supplementary Fig. S33).

##### Quality of life

One trial, randomising a total of 208 participants, reported on the quality of life by using the EuroQol-5D questionnaire at both six weeks and three months postoperative^64^ (33 participants comprising 14 in the experimental group and 19 in the control group, were considered lost to follow-up due to no reporting of outcome date). Experimental versus control did not affect the median EuroQol-5D single summary index score (experimental 0.80, interquartile range (IQR) 0.73–1.00, *n* = 88 versus control 0.88, IQR 0.76–1.00, *n* = 87). This result was considered at high risk of bias (Appendix C: Characteristics of trials, data extraction and risk of bias).

##### Brain damage on imaging at maximal follow-up

Six trials randomising a total of 754 participants reported an outcome that was classified as brain damage on imaging (38 participants comprising 18 in the experimental group and 20 in the control group, were considered lost to follow-up due to no reporting of outcome data). The classified outcomes included stroke visualised on imaging from 5 days to 3 months postoperative,^44,49,55,64^ and brain injury on cranial ultrasound scans up to term age.^60,61^ In the experimental group, 65/354 (18.4%) suffered an event, versus 60/362 (16.6%) in the control group. Meta-analysis showed no significant difference on the outcome (RR 1.10, 95% CI 0.90–1.34; *p* = 0.80; *I*^2^ = 0%; 716 participants; six trials, Appendix D, Supplementary Fig. S35). This result was considered at high risk of bias (Appendix C: Characteristics of trials, data extraction and risk of bias assessments).

##### Proportion of participants with one or more adverse events

Five trials, randomising a total of 734 participants reported one or more outcomes classified as adverse events (13 participants comprising six in the experimental group and 7 in the control group, were considered lost to follow-up due to no reporting on adverse events). For an overview of the events reported in each trial, see ‘Appendix C: Characteristics of trials, data extraction and risk of bias assessment’. In the experimental group, 79/362 (21.8%) participants experienced one or more adverse events, versus 104/359 (30.0%) in the control group. The primary analysis (random-effects meta-analysis) showed no significant difference between the experimental and control group (RR 0.75, 95% CI 0.55–1.03; *p* = 0.08; *I*^2^ = 27.3%; 721 participants; five trials) (Appendix D, Supplementary Fig. S27). This result was considered at high risk of bias (see Appendix C: Characteristics of trials, data extraction and risk of bias assessments). There was no significant heterogeneity (*I*^2^ = 27.3%; *p* = 0.24).

#### Exploratory outcomes

##### Any evidence of a negative impact on the brain

Seventeen trials randomising a total of 2169 participants, reported an outcome that was classified as any evidence of a negative impact on the brain (76 participants comprising 45 in the experimental group and 31 in the control group, were considered lost to follow-up due to no reporting of outcome data). The classified outcomes included postoperative cognitive impairment 5–7 days postoperative (in one trial, assessment time was uncertain),^42,46,65^ postoperative cognitive decline 2–7 days postoperative^48,54,57^ postoperative cognitive dysfunction 7 days postoperative,^50^ postoperative delirium 7–30 days postoperative or discharge,^44,47,63^ short-term postoperative neurologic deficit (assessment time is uncertain),^59^ stroke 5–30 days postoperative (in one trial, assessment time was uncertain),^49,55,64^ brain injury on cranial ultrasound up to term age,^60^ abnormal general movements at discharge or term age,^61^ or unfavourable Glasgow outcome score of 2 or 3 at discharge.^62^ In the experimental group, 231/1079 (21.4%) suffered an event versus 323/1014 (31.8%) in the control group. Meta-analysis showed a significant difference on the outcome (RR 0.67, 95% CI 0.52–0.87; *p* = < 0.01; *I*^2^ = 60.6%; 2093 participants; 17 trials, Appendix D, Supplementary Fig. S39). This result was considered at high risk of bias (Appendix C: Characteristics of trials, data extraction and risk of bias). Testing for statistical heterogeneity was significant (*I*^2^ = 60.6%, *p* ≤ 0.01). Based on visual inspection of the forest plot, the results from Mohandas et al.^48^ was suspected for being the main reason for heterogeneity. However, after excluding Mohandas et al. in a sensitivity analysis, heterogeneity was still statistically significant (*I*^2^ = 42.3%, *p* = 0.03) (Appendix D, Supplementary Fig. S40).

##### Individual serious adverse events and adverse events

Due to inconsistency in the definition and reporting of serious and non-serious adverse events, we decided post hoc not to analyse individual events. Instead, a full overview on reported individual events can be found in ‘Appendix C: Characteristics of trials, data extraction, and risk of bias assessment’.

##### Differences in the methodology between protocol and review

Due to limited time, we used a modified and simpler search strategy (see ‘Search strategy, study selection and data extraction’) than described in the protocol.^19^ As mentioned under ‘Search strategy, study selection and data extraction’, only one author (MLH) conducted the literature search and study selection, instead of two authors (MLH and SH-S). As the secondary and exploratory outcomes were only hypothesis generating, and since trials from multiple subgroups were identified, we decided post hoc only to conduct GRADE assessment and present a summary of findings table based on the three primary outcomes. In addition, subgroup analyses and TSA-adjusted CIs were only calculated and reported for the primary outcomes. Risk of bias assessments were still conducted for all outcomes.

The literature search and study selection were conducted by MLH who, if in doubt regarding the eligibility of studies, consulted with GG or JCJ.

## Discussion

To our knowledge, this review is the first to assess the effects of clinical care with access to cerebral NIRS monitoring versus clinical care without access to cerebral NIRS monitoring on a variety of clinical outcomes, by pooling and meta-analysing data from trials across various clinical settings. We included 25 trials, randomising a total of 2606 children and adults to clinical care with and without cerebral NIRS monitoring. The clinical settings included cardiac surgery,^42,44–49,51,52,64,65^ non-cardiac surgery,^53–59,63,66^ neonatal intensive care,^60,61^ and neurocritical care of patients with traumatic brain injury.^62^ Meta-analysis and TSA demonstrated that the obtained information size was insufficient to detect or reject that adding cerebral NIRS monitoring to clinical care, decreases the risk of death; moderate or severe, persistent cognitive or neurological deficit, significantly affecting daily life; or experiencing one or more serious adverse events.^19^ The primary analyses of the secondary outcome ‘mild, moderate or severe, temporary or persistent, cognitive or neurologic deficit’, and the exploratory outcome ‘any negative impact on the brain’ showed a significant difference between the experimental and control group, in favour of the intervention. For the secondary outcome ‘mild, moderate or severe, temporary or persistent, cognitive or neurological deficit’, after the sensitivity analysis excluding Mohandas et al.,^48^ heterogeneity was insignificant and the meta-analysis still showed a significant difference in the outcome with a relative risk of 0.72 (95% CI 0.60–0.85). Although these results might seem promising in terms of the potential benefit of adding cerebral NIRS monitoring to clinical care, it is important to emphasise that these outcomes were predefined as hypothesis-generating^19^ and the results should be interpreted as such. To avoid multiplicity issues, no subgroup analyses on these outcomes were conducted.^18^ In addition, the results were at high risk of bias.

This review has several strengths. First, the methodology is described in detail in our published protocol which decreases the risk of outcome reporting bias.^68^ Second, we minimised the risk of random errors by using the eight-step procedure by Jakobsen et al.^18^ to assess significance for the primary outcomes. This included adjusting the threshold (*p* value) for statistical significance,^69^ calculating Bayes factor,^31^ and conducting TSA.^17^ We choose to apply TSA to control the risks of false positive and false negative conclusions. The boundaries were defined by, among other parameters, a predicted relative risk effect size of 20%, since there is little reason to predict that the effect would be larger than that. In fact, the effect could turn out to be smaller or non-existent. Third, all trials and their reported outcomes underwent risk of bias assessment, according to the Risk of Bias 2 tool, to quantify the risk of systematic errors. Fourth, despite pooling data across different clinical settings, statistical heterogeneity was insignificant for two out of three primary outcomes. Thus, study interpretation was not substantially impacted by heterogeneity. This supports that classifying and pooling outcomes from trials across different clinical settings in this meta-analysis was a valid approach.

This review also has several limitations. First, the search strategy was modified due to time constraints among authors and relevant trials might therefore be missing in the present review. However, we judged it highly unlikely that complying with the protocol in this respect would substantially alter the finding of the review. Despite including 25 trials, we did not reach a sufficient information size, as indicated by TSA, for any of the three primary outcomes. As several trials evaluating the use of cerebral NIRS monitoring in various clinical settings are ongoing, the accrued information size might increase in the future.^70–75^ In addition, all trials were assessed as high risk of bias and only two trials reported an outcome that was classified at low risk of bias. Thus, the reported trials demonstrate a high risk of having underestimated potential harms and overestimated potential benefits.^76^

Finally, why is it that 20 years after the introduction of the technology, the evidence from randomised trials is still so limited? First, compared to new drug therapies, for medical devices, there are no legal requirements for demonstration of clinical benefit. Second, intensive care has in general developed by mechanistic reasoning, solving the many problems of organ failure with partial and short-sighted goals of improvement in biomarkers. And overall, this has been successful with steadily improved chances of survival of steadily more fragile and complex medical and surgical patients. Third, the ethical and legal overhead on randomised clinical trials makes it tempting to shortcut the paved way to evidence-based practice.

## Conclusions

Due to an insufficient information size and high risk of bias in reported trials, the evidence on the effects of clinical care with access to cerebral NIRS monitoring versus clinical care without access to cerebral NIRS monitoring is very uncertain. To increase certainty, additional large-scale trials, focusing on lowering their risk of bias, are needed.

## Supplementary information

Electronic supplementary material
