# Supplementary material for: Cerebral near-infrared spectroscopy monitoring (NIRS) in children and adults: a systematic review with meta-analysis
Source: Pediatr Res. 2022 Feb 22;96(4):856–67. doi: 10.1038/s41390-022-01995-z (PMC11502593; doi:10.1038/s41390-022-01995-z)
Supplement: Supplementary file 1 — Electronic supplementary material [file 41390_2022_1995_MOESM1_ESM.pdf]

Electronic supplementary material

Supplement to

The clinical effect of cerebral near-infrared spectroscopy monitoring (NIRS) in children and adults: a systematic review with meta-analysis and Trial Sequential Analysis

Hansen et. al

Appendix A: PRISMA Checklist……………………………….p 2 to 3

Appendix B: Search strategy…………………………………....p 4 to 4

Appendix C: Characteristics of trials, data extraction and risk of bias assessment…………………………………………………………..p 5 to 5

Appendix D: Statistical analyses………………………………..p 6 to 41

Appendix E: Datafile for statistical analysis program………...p 42 to 42

Appendix F: Synthax for STATA/SE 17.0……………………..p 43 to 43

Appendix G: Correspondence with trialists……………………p 44 to 44

Appendix A: PRISMA checklist

Section/topic	#	Checklist item	Reported on page #
**TITLE**			
Title	1	Identify the report as a systematic review, meta-analysis, or both.	1
**ABSTRACT**			
Structured summary	2	Provide a structured summary including, as applicable: background; objectives; data sources; study eligibility criteria, participants, and interventions; study appraisal and synthesis methods; results; limitations; conclusions and implications of key findings; systematic review registration number.	5
**INTRODUCTION**			
Rationale	3	Describe the rationale for the review in the context of what is already known.	6-7
Objectives	4	Provide an explicit statement of questions being addressed with reference to participants, interventions, comparisons, outcomes, and study design (PICOS).	7
**METHODS**			
Protocol and registration	5	Indicate if a review protocol exists, if and where it can be accessed (e.g., Web address), and, if available, provide registration information including registration number.	7
Eligibility criteria	6	Specify study characteristics (e.g., PICOS, length of follow-up) and report characteristics (e.g., years considered, language, publication status) used as criteria for eligibility, giving rationale.	7-8
Information sources	7	Describe all information sources (e.g., databases with dates of coverage, contact with study authors to identify additional studies) in the search and date last searched.	9
Search	8	Present full electronic search strategy for at least one database, including any limits used, such that it could be repeated.	9 (and appendix)
Study selection	9	State the process for selecting studies (i.e., screening, eligibility, included in systematic review, and, if applicable, included in the meta-analysis).	9
Data collection process	10	Describe method of data extraction from reports (e.g., piloted forms, independently, in duplicate) and any processes for obtaining and confirming data from investigators.	9
Data items	11	List and define all variables for which data were sought (e.g., PICOS, funding sources) and any assumptions and simplifications made.	9-10 (and appendix)
Risk of bias in individual studies	12	Describe methods used for assessing risk of bias of individual studies (including specification of whether this was done at the study or outcome level), and how this information is to be used in any data synthesis.	10
Summary measures	13	State the principal summary measures (e.g., risk ratio, difference in means).	10-11
Synthesis of results	14	Describe the methods of handling data and combining results of studies, if done, including measures of consistency (e.g., I^2) for each meta-analysis.	10-11

Risk of bias across studies	15	Specify any assessment of risk of bias that may affect the cumulative evidence (e.g., publication bias, selective reporting within studies).	11
Additional analyses	16	Describe methods of additional analyses (e.g., sensitivity or subgroup analyses, meta-regression), if done, indicating which were pre-specified.	11
**RESULTS**			
Study selection	17	Give numbers of studies screened, assessed for eligibility, and included in the review, with reasons for exclusions at each stage, ideally with a flow diagram.	11-14
Study characteristics	18	For each study, present characteristics for which data were extracted (e.g., study size, PICOS, follow-up period) and provide the citations.	11-14 (and appendix)
Risk of bias within studies	19	Present data on risk of bias of each study and, if available, any outcome level assessment (see item 12).	14-20 (and appendix)
Results of individual studies	20	For all outcomes considered (benefits or harms), present, for each study: (a) simple summary data for each intervention group (b) effect estimates and confidence intervals, ideally with a forest plot.	14-20. Data extraction for individ. studies in appendix
Synthesis of results	21	Present results of each meta-analysis done, including confidence intervals and measures of consistency.	14-20
Risk of bias across studies	22	Present results of any assessment of risk of bias across studies (see Item 15).	14-20 (and appendix)
Additional analysis	23	Give results of additional analyses, if done (e.g., sensitivity or subgroup analyses, meta-regression [see Item 16]).	14-20 (and appendix
**DISCUSSION**			
Summary of evidence	24	Summarize the main findings including the strength of evidence for each main outcome; consider their relevance to key groups (e.g., healthcare providers, users, and policy makers).	20-22
Limitations	25	Discuss limitations at study and outcome level (e.g., risk of bias), and at review-level (e.g., incomplete retrieval of identified research, reporting bias).	20-22
Conclusions	26	Provide a general interpretation of the results in the context of other evidence, and implications for future research.	23
**FUNDING**			
Funding	27	Describe sources of funding for the systematic review and other support (e.g., supply of data); role of funders for the systematic review.	3

Appendix B: Search strategy

**PubMed (November 2020)**
(((((((((((((cerebral oxygen) OR (cerebral oximetry)) OR (cerebral saturation)) OR (cerebral oxymeter)) OR (cerebral tissue oxygen)) OR (near-infrared spectroscopy)) OR (cerebral oxygenation)) OR (brain oxygen)) OR (cerebral NIRS)) OR (near-infrared refracted spectroscopy)) OR (cerebral near infrared spectroscopy)) OR (near infrared spectroscopy)) AND ((((((randomized controlled trial) OR (randomised controlled trial)) OR (randomized clinical trial)) OR (randomised clinical trial)) OR (randomized)) OR (randomised))) AND ((((((randomized controlled trial) OR (randomised controlled trial)) OR (randomized clinical trial)) OR (randomised clinical trial)) OR (randomized)) OR (randomised))
**MEDLINE (Ovid) (March 2021)**
1. exp Spectroscopy, Near-Infrared/<br>2. (((near adj infrared) or nir) and (spectroscop* or spectromet*)).ti,ab.<br>3. 1 or 2<br>4. exp Cerebrovascular Circulation/<br>5. exp Oximetry/<br>6. ((cerebr* and (nir* or oxygen* or saturation* or oximetr* or perfusion*)) or (oxygen and (brain or saturation*))).ti,ab.<br>7. 4 or 5 or 6<br>8. 3 or 7<br>9. (randomized controlled trial or controlled clinical trial).pt. or clinical trials as topic.sh. or trial.ti.<br>10. (random* or blind*).ti,ab.<br>11. 8 and (9 or 10)<br>12. 11 not (exp animals/ not humans.sh.)

Appendix C: Characteristics of trials, data extraction and risk of bias assessment

Please go to

LINK FIGSHARE: https://figshare.com/s/a9ef63687962c4e686a8

For full access to extracted data, risk of bias assessment and characteristics of included and excluded trials.

Appendix D: Statistical analyses

Content declaration

All-cause mortality 8
Primary analysis 8
Subgroup analyses 9
Clinical setting (cardiac surgery compared to neonatal intensive care compared to neurocritical care compared to non-cardiac surgery) 9
Industry funding (funding not reported compared to industry funded compared to no industry funding) 10
Cerebral NIRS monitoring in the control group (blinded cerebral NIRS monitoring in the control group compared to no cerebral NIRS monitoring in the control group) 11
Trial Sequential Analysis 12
Bayes factor 12
Sensitivity analyses 13
Best-worst case analysis 13
Worst-best case analysis 13
Funnel plot 14
Moderate or severe, persistent, cognitive or neurological deficit, at maximal follow-up 15
Primary analysis 15
Subgroup analyses 16
Risk of bias (high compared to low) 16
Clinical setting (cardiac surgery compared to neonatal intensive care compared to neurocritical care compared to non-cardiac surgery) 17
Industry funding (funding not reported compared to industry funded compared to no industry funding) 18
Cerebral NIRS monitoring in the control group (blinded cerebral NIRS monitoring in the control group compared to no cerebral NIRS monitoring in the control group) 19
Trial Sequential Analysis 20
Bayes factor 20
Sensitivity analyses 21
Best-worst case analysis 21
Worst-best case analysis 21
Heterogeneity (excluding Mohandas et al 2013) 22
Proportion of participants with one or more serious adverse events 23
Primary analysis 23

- **Subgroup analyses** ... 25
  - **Risk of bias (high compared to low)** ... 25
  - **Clinical setting (cardiac surgery compared to neonatal intensive care compared to neurocritical care compared to non-cardiac surgery)** ... 26
  - **Industry funding (funding not reported compared to industry funded compared to no industry funding)** ... 27
  - **Cerebral NIRS monitoring in the control group (blinded cerebral NIRS monitoring in the control group compared to no cerebral NIRS monitoring in the control group)** ... 28
- **Trial Sequential Analysis** ... 29
- **Bayes factor** ... 29
- **Sensitivity analyses** ... 30
  - **Best-worst case analysis** ... 30
  - **Worst-best case analysis** ... 31
  - **Heterogeneity (excluding Mohandas et al. 2013)** ... 32
- **Funnel plot** ... 33

Mild, moderate or severe, temporary or persistent, cognitive or neurological deficit ... 34
- **Primary analysis** ... 34
- **Sensitivity analysis** ... 36
  - **Heterogeneity (excluding Mohandas et al. 2013)** ... 36

Brain damage on imaging at maximal follow-up ... 37
- **Primary analysis** ... 37

Proportion of participants with one or more adverse events ... 38
- **Primary analysis** ... 38

Any evidence of a negative impact on the brain ... 39
- **Primary analysis** ... 39
- **Sensitivity analysis** ... 41
  - **Heterogeneity (excluding Mohandas et al. 2013)** ... 41

All-cause mortality

Primary analysis

Figure S1 – fixed effect

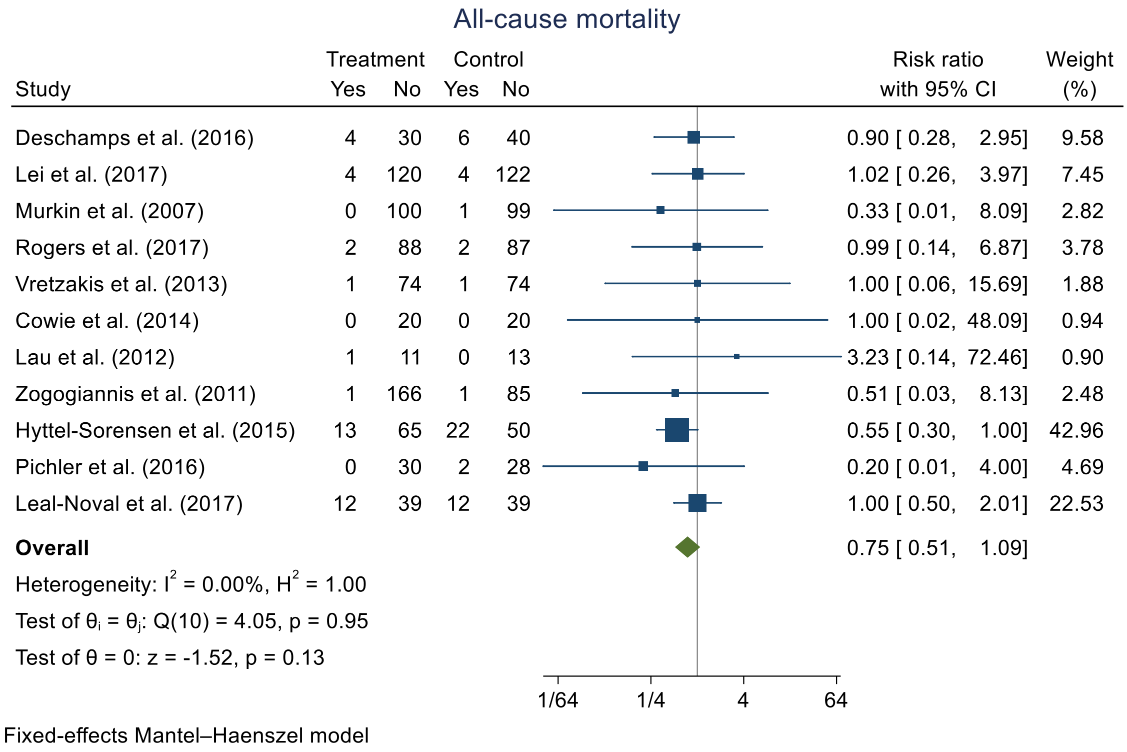

Figure S2 – random effects

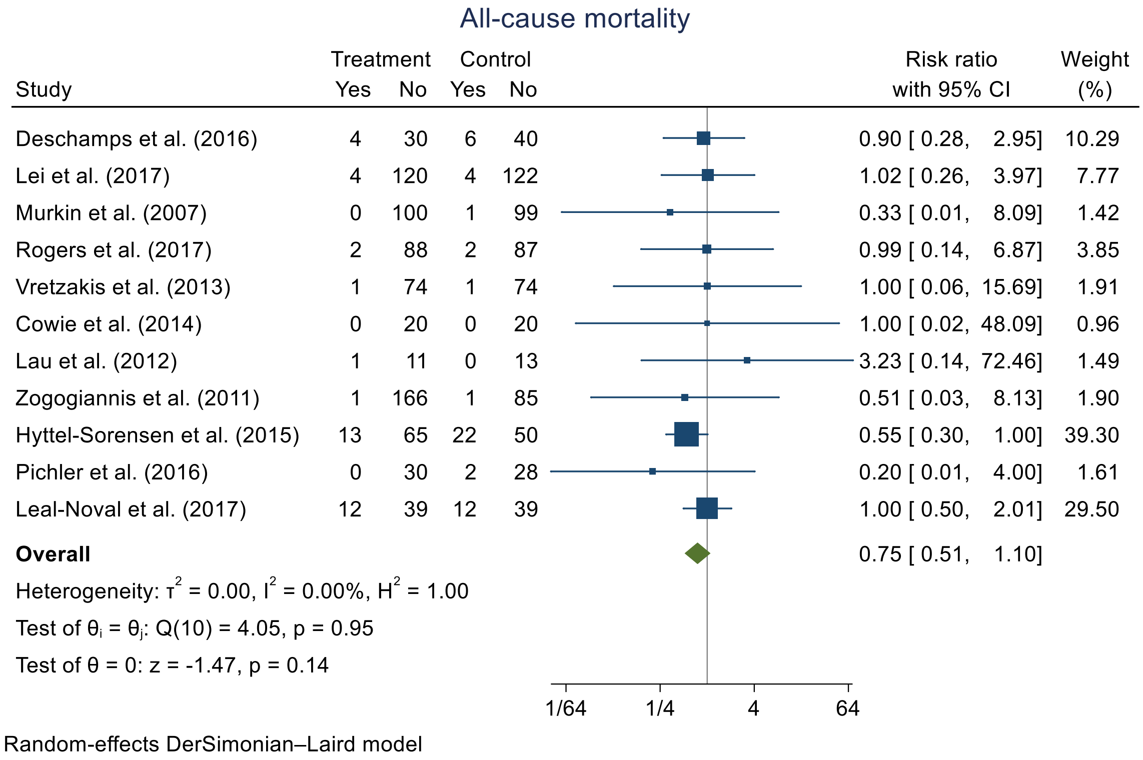

Subgroup analyses

Clinical setting (cardiac surgery compared to neonatal intensive care compared to neurocritical care compared to non-cardiac surgery)

Figure S3

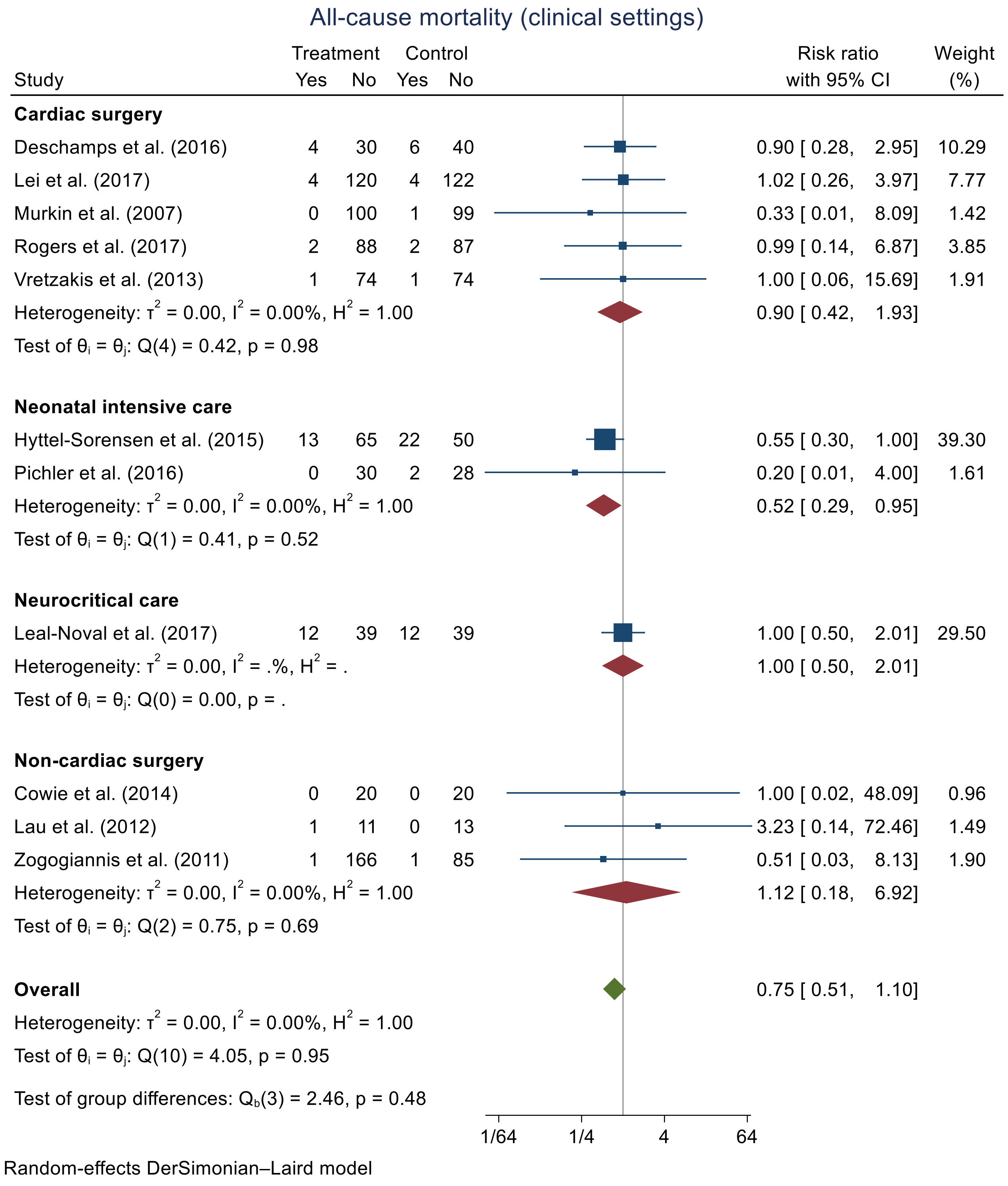

Industry funding (funding not reported compared to industry funded compared to no industry funding)

Figure S4

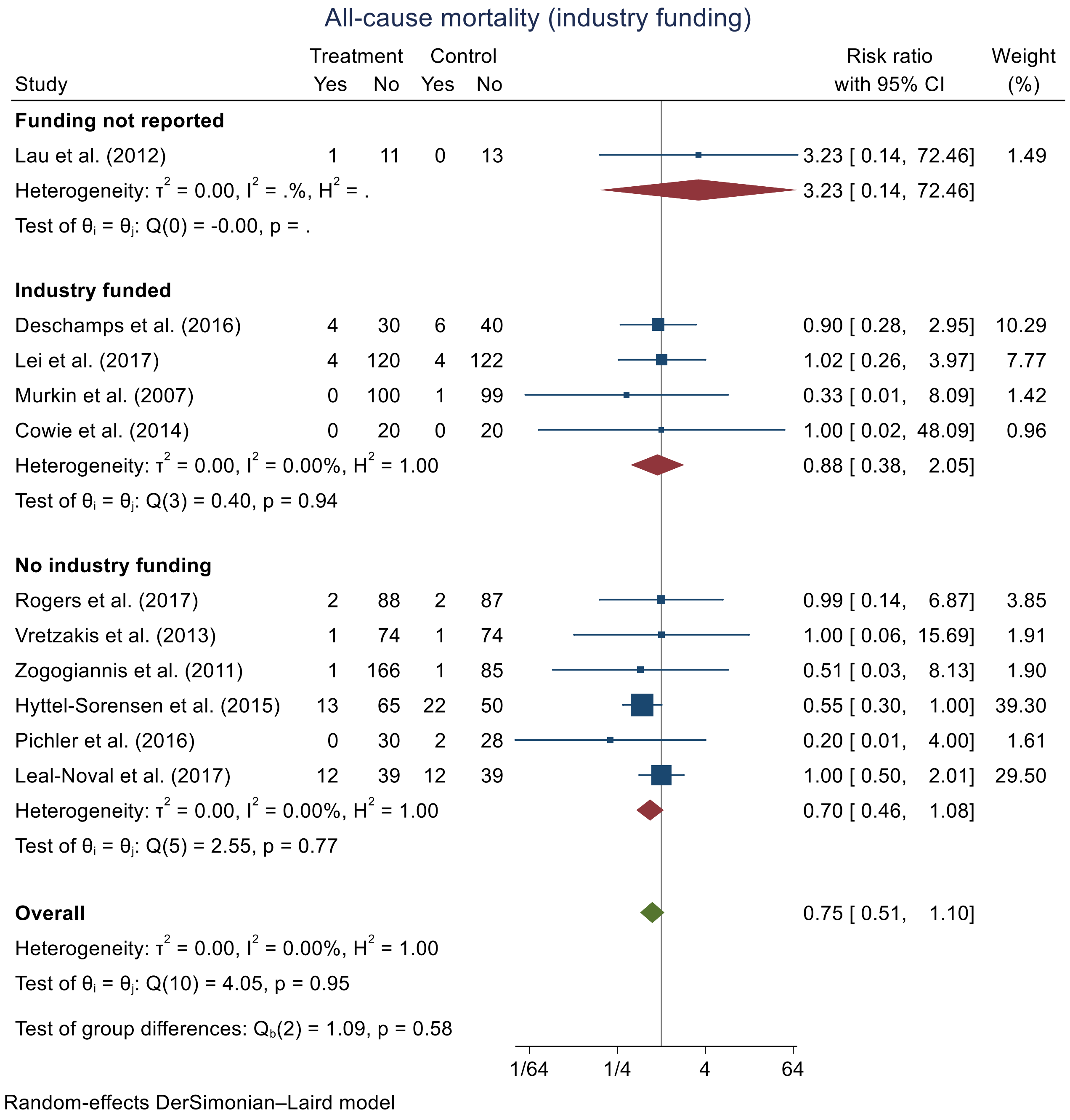

Cerebral NIRS monitoring in the control group (blinded cerebral NIRS monitoring in the control group compared to no cerebral NIRS monitoring in the control group)

Figure S5

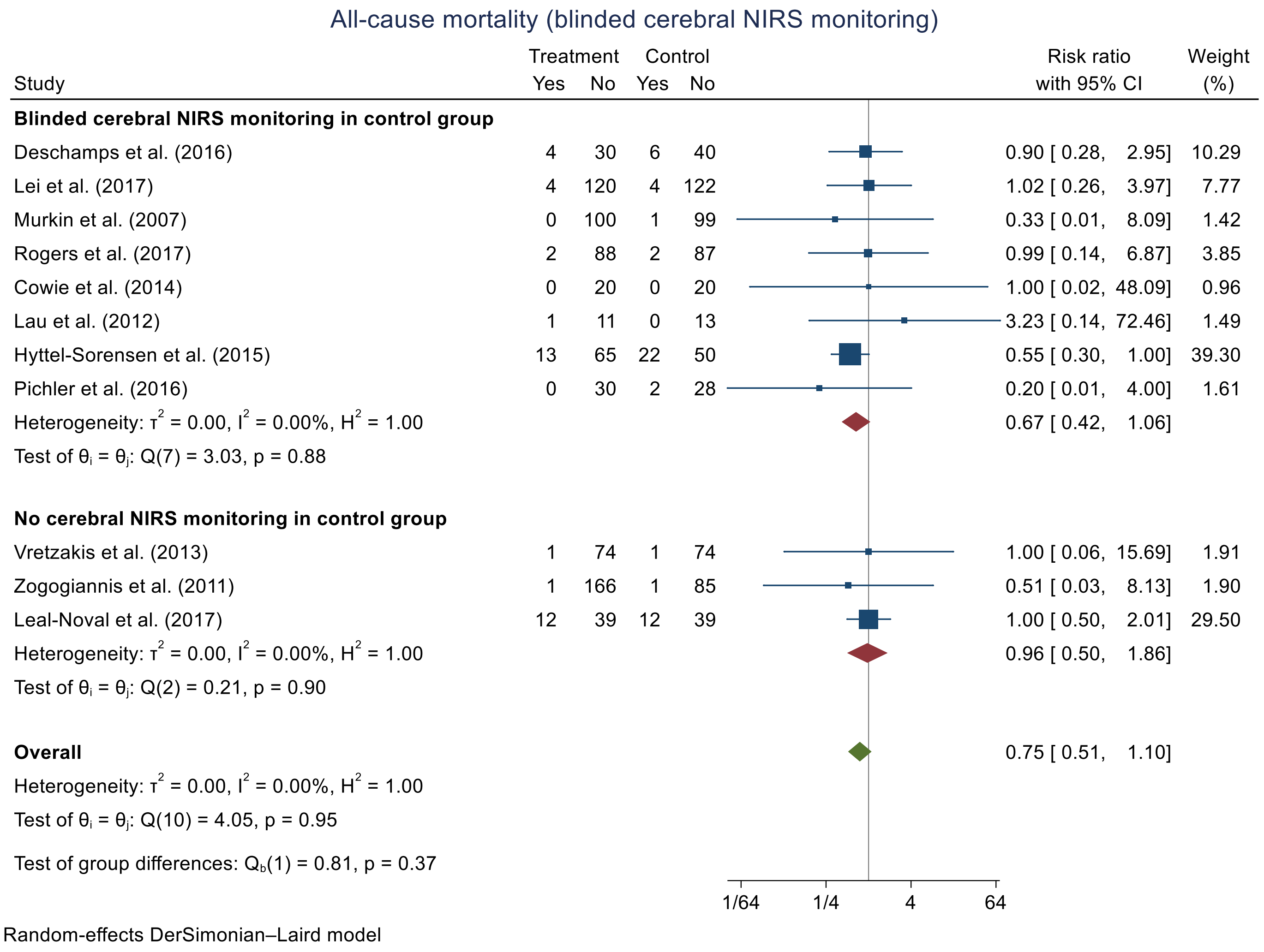

Trial Sequential Analysis

Figure S6 – TSA all-cause mortality

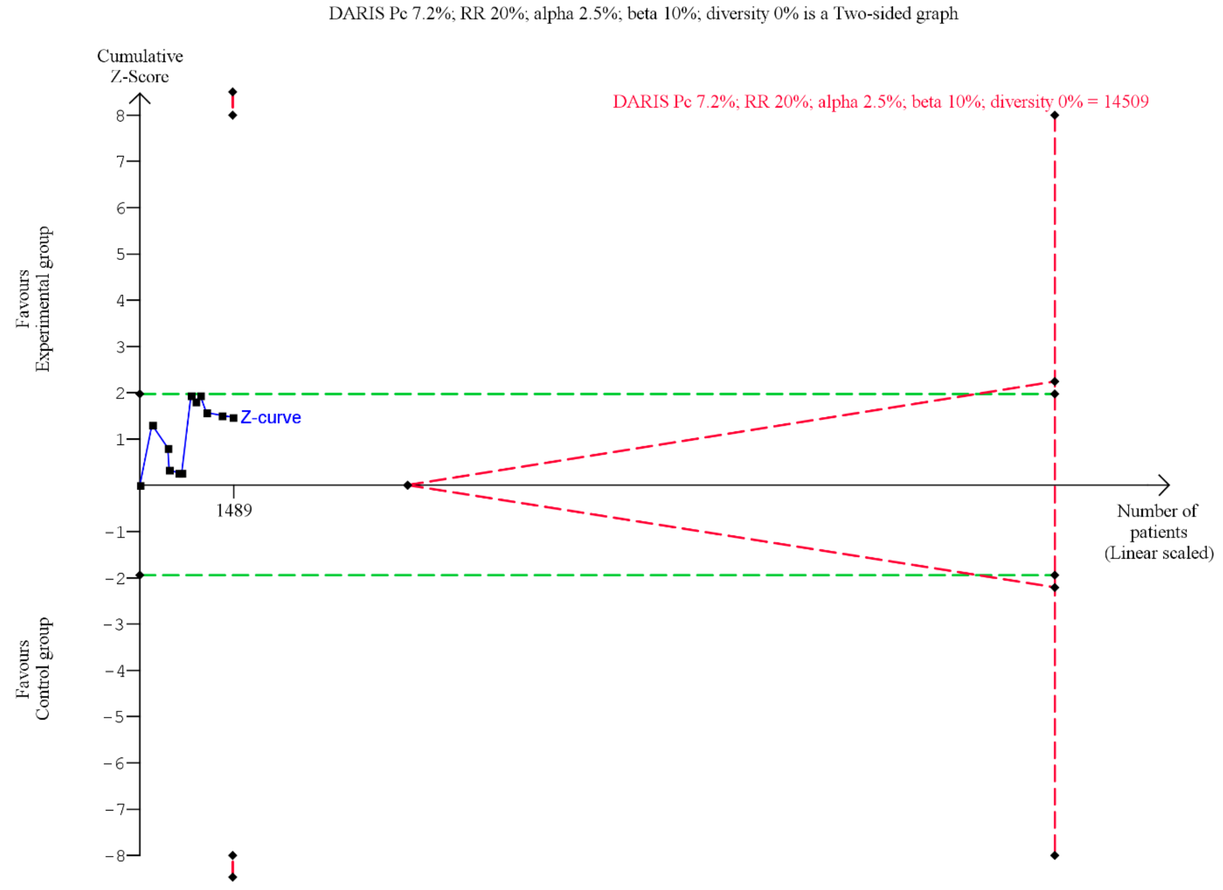

Trial Sequential analysis adjusted CI: 0.16 to 3.62 (p=0.1457), diversity = 0%

Bayes factor

LnRR meta-analysis = -0,29
LnSE meta-analysis = 0.20
LnRR a priori = -0.22

Bayes factor = 0.37

Sensitivity analyses

Best-worst case analysis

Assuming a beneficial event in the experimental group and harmful in the control group

Figure S7

All-cause mortality (best-worst case analysis)

Study	Treatment Yes	Treatment No	Control Yes	Control No	Risk ratio with 95% CI	Weight (%)
Deschamps et al. (2016)	4	30	6	40	0.90 [0.28, 2.95]	12.32
Lei et al. (2017)	4	120	4	122	1.02 [0.26, 3.97]	9.85
Murkin et al. (2007)	0	100	1	99	0.33 [0.01, 8.09]	2.11
Rogers et al. (2017)	2	100	19	87	0.11 [0.03, 0.46]	9.09
Vretzakis et al. (2013)	1	74	1	74	1.00 [0.06, 15.69]	2.80
Cowie et al. (2014)	0	20	0	20	1.00 [0.02, 48.09]	1.45
Lau et al. (2012)	1	11	0	13	3.23 [0.14, 72.46]	2.22
Zogogiannis et al. (2011)	1	166	1	85	0.51 [0.03, 8.13]	2.79
Hyttel-Sorensen et al. (2015)	13	73	30	50	0.40 [0.23, 0.72]	30.14
Pichler et al. (2016)	0	30	2	28	0.20 [0.01, 4.00]	2.38
Leal-Noval et al. (2017)	12	39	12	39	1.00 [0.50, 2.01]	24.83
**Overall**					0.58 [0.36, 0.93]	

Heterogeneity: $\tau^2 = 0.11$, $I^2 = 18.48\%$, $H^2 = 1.23$

Test of $\theta_i = \theta_j$: $Q(10) = 12.27$, $p = 0.27$

Test of $\theta = 0$: $z = -2.24$, $p = 0.03$

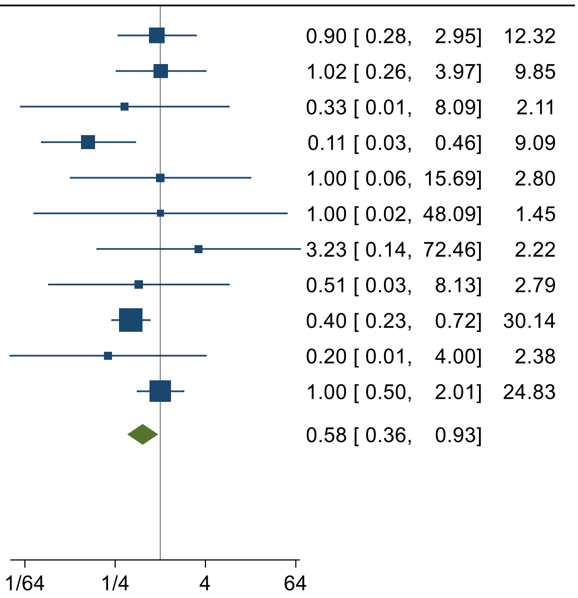

Random-effects DerSimonian–Laird model

Worst-best case analysis

Assuming a harmful event in the experimental group and beneficial in the control group

Figure S8

All-cause mortality (worst-best case analysis)

Study	Treatment Yes	Treatment No	Control Yes	Control No	Risk ratio with 95% CI	Weight (%)
Deschamps et al. (2016)	4	30	6	40	0.90 [0.28, 2.95]	8.70
Lei et al. (2017)	4	120	4	122	1.02 [0.26, 3.97]	6.57
Murkin et al. (2007)	0	100	1	99	0.33 [0.01, 8.09]	1.20
Rogers et al. (2017)	14	88	2	104	7.27 [1.70, 31.21]	5.76
Vretzakis et al. (2013)	1	74	1	74	1.00 [0.06, 15.69]	1.61
Cowie et al. (2014)	0	20	0	20	1.00 [0.02, 48.09]	0.81
Lau et al. (2012)	1	11	0	13	3.23 [0.14, 72.46]	1.26
Zogogiannis et al. (2011)	1	166	1	85	0.51 [0.03, 8.13]	1.60
Hyttel-Sorensen et al. (2015)	21	65	22	58	0.89 [0.53, 1.49]	46.14
Pichler et al. (2016)	0	30	2	28	0.20 [0.01, 4.00]	1.36
Leal-Noval et al. (2017)	12	39	12	39	1.00 [0.50, 2.01]	24.96
**Overall**					1.02 [0.72, 1.45]	

Heterogeneity: $\tau^2 = 0.00$, $I^2 = 0.00\%$, $H^2 = 1.00$

Test of $\theta_i = \theta_j$: $Q(10) = 9.69$, $p = 0.47$

Test of $\theta = 0$: $z = 0.12$, $p = 0.91$

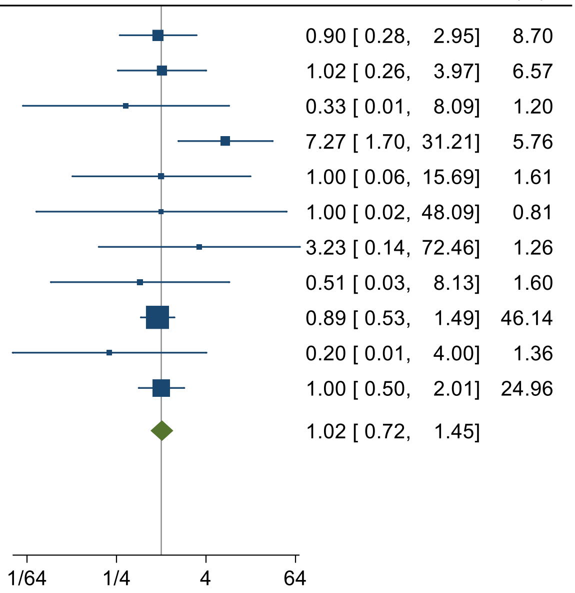

Random-effects DerSimonian–Laird model

Funnel plot

Figure S9

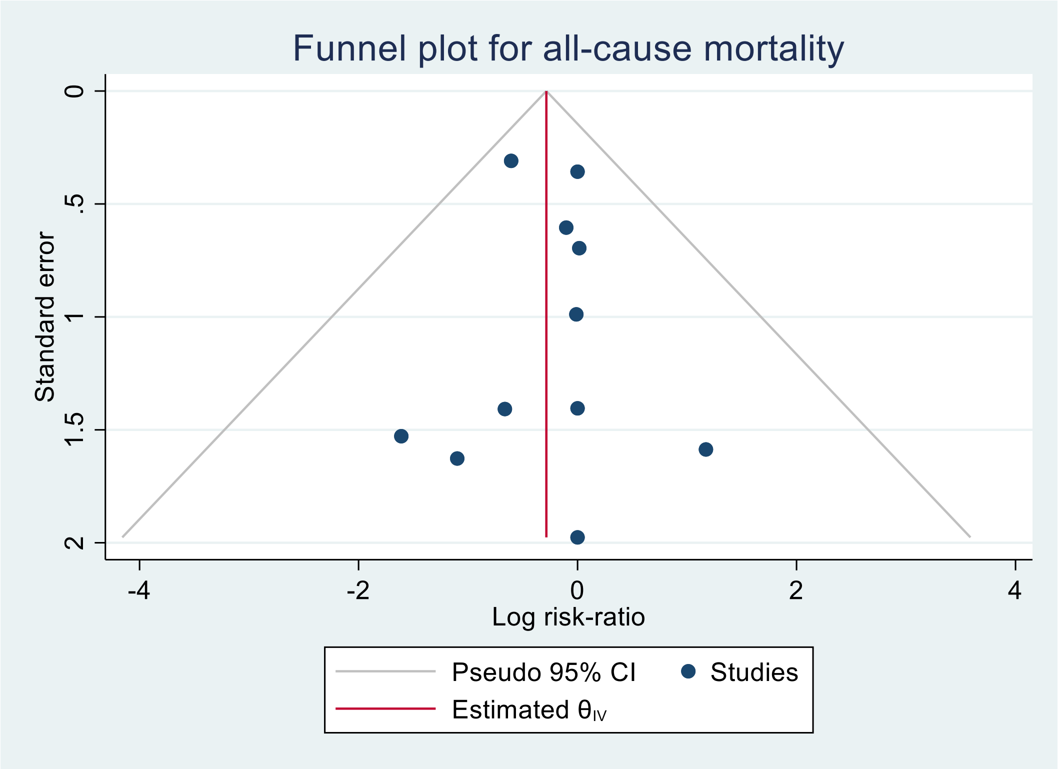

Moderate or severe, persistent, cognitive or neurological deficit, at maximal follow-up

Primary analysis

Figure S10 – fixed effect

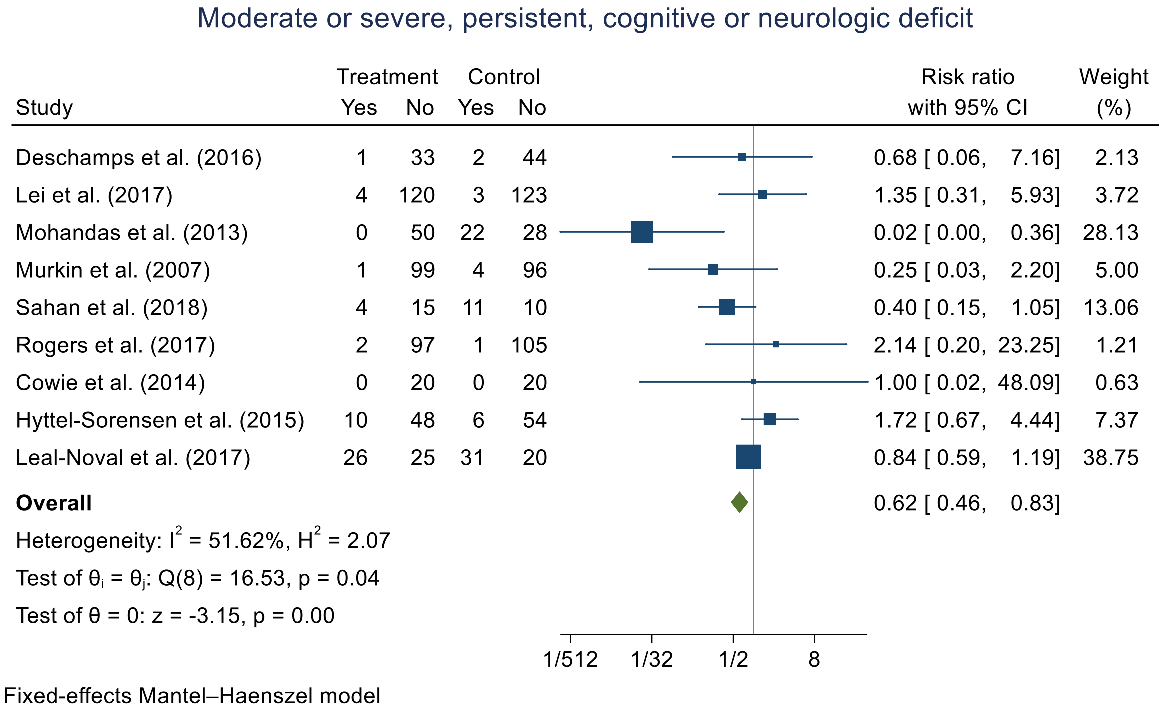

Figure S11 – random effects

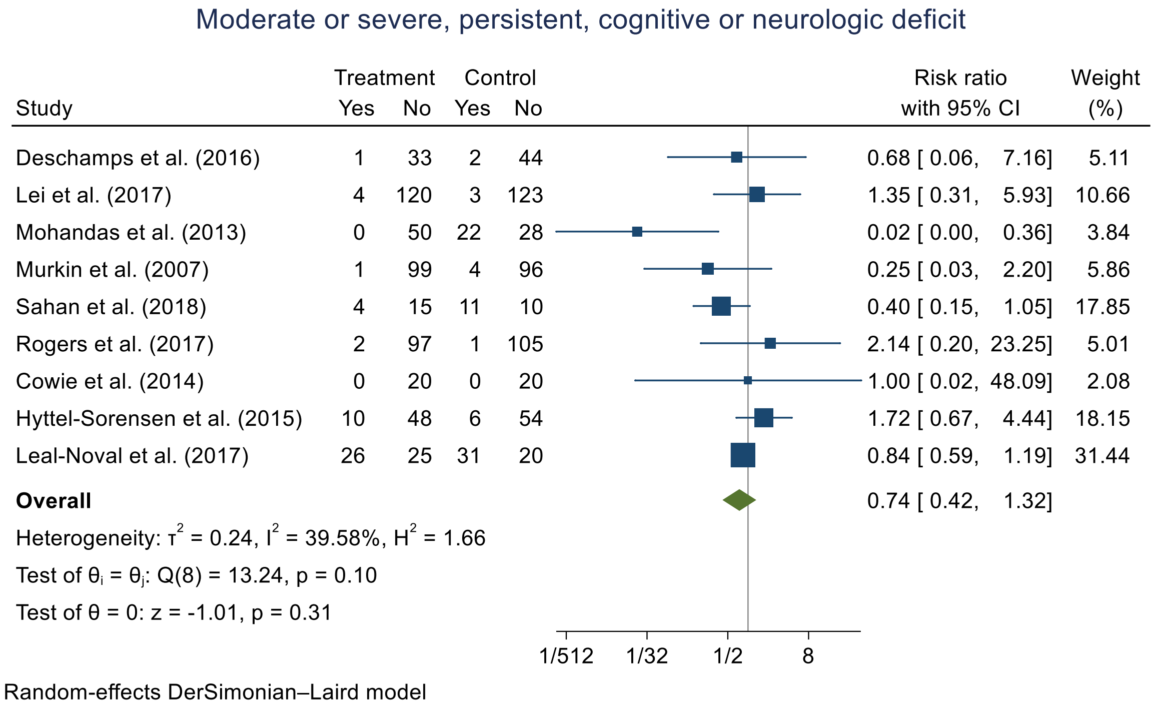

Subgroup analyses

Risk of bias (high compared to low)

Figure S12

Moderate or severe, persistent, cognitive or neurological deficit (risk of bias)

Study	Treatment Yes	Treatment No	Control Yes	Control No	Risk ratio with 95% CI	Weight (%)
**High risk of bias**						
Deschamps et al. (2016)	1	33	2	44	0.68 [0.06, 7.16]	5.11
Lei et al. (2017)	4	120	3	123	1.35 [0.31, 5.93]	10.66
Mohandas et al. (2013)	0	50	22	28	0.02 [0.00, 0.36]	3.84
Murkin et al. (2007)	1	99	4	96	0.25 [0.03, 2.20]	5.86
Sahan et al. (2018)	4	15	11	10	0.40 [0.15, 1.05]	17.85
Cowie et al. (2014)	0	20	0	20	1.00 [0.02, 48.09]	2.08
Hyttel-Sorensen et al. (2015)	10	48	6	54	1.72 [0.67, 4.44]	18.15
Leal-Noval et al. (2017)	26	25	31	20	0.84 [0.59, 1.19]	31.44
Heterogeneity: $\tau^2 = 0.27$, $I^2 = 44.44\%$, $H^2 = 1.80$					0.70 [0.38, 1.28]	
Test of $\theta_i = \theta_j$: Q(7) = 12.60, p = 0.08						
**Low risk of bias**						
Rogers et al. (2017)	2	97	1	105	2.14 [0.20, 23.25]	5.01
Heterogeneity: $\tau^2 = 0.00$, $I^2 = .\%$, $H^2 = .$					2.14 [0.20, 23.25]	
Test of $\theta_i = \theta_j$: Q(0) = -0.00, p = .						
**Overall**					0.74 [0.42, 1.32]	
Heterogeneity: $\tau^2 = 0.24$, $I^2 = 39.58\%$, $H^2 = 1.66$						
Test of $\theta_i = \theta_j$: Q(8) = 13.24, p = 0.10						
Test of group differences: $Q_b(1) = 0.80$, p = 0.37						

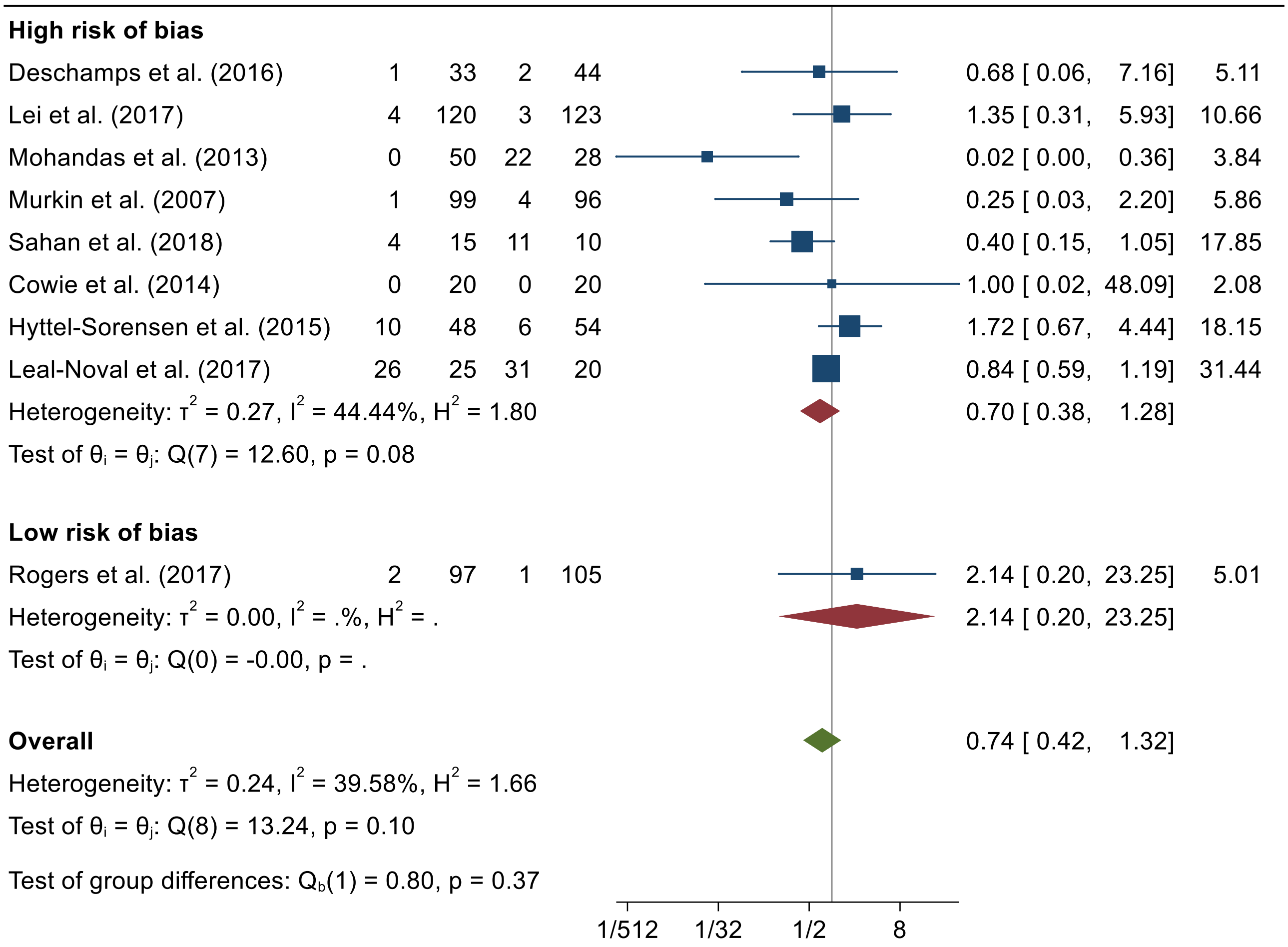

Random-effects DerSimonian–Laird model

Clinical setting (cardiac surgery compared to neonatal intensive care compared to neurocritical care compared to non-cardiac surgery)
Figure S13

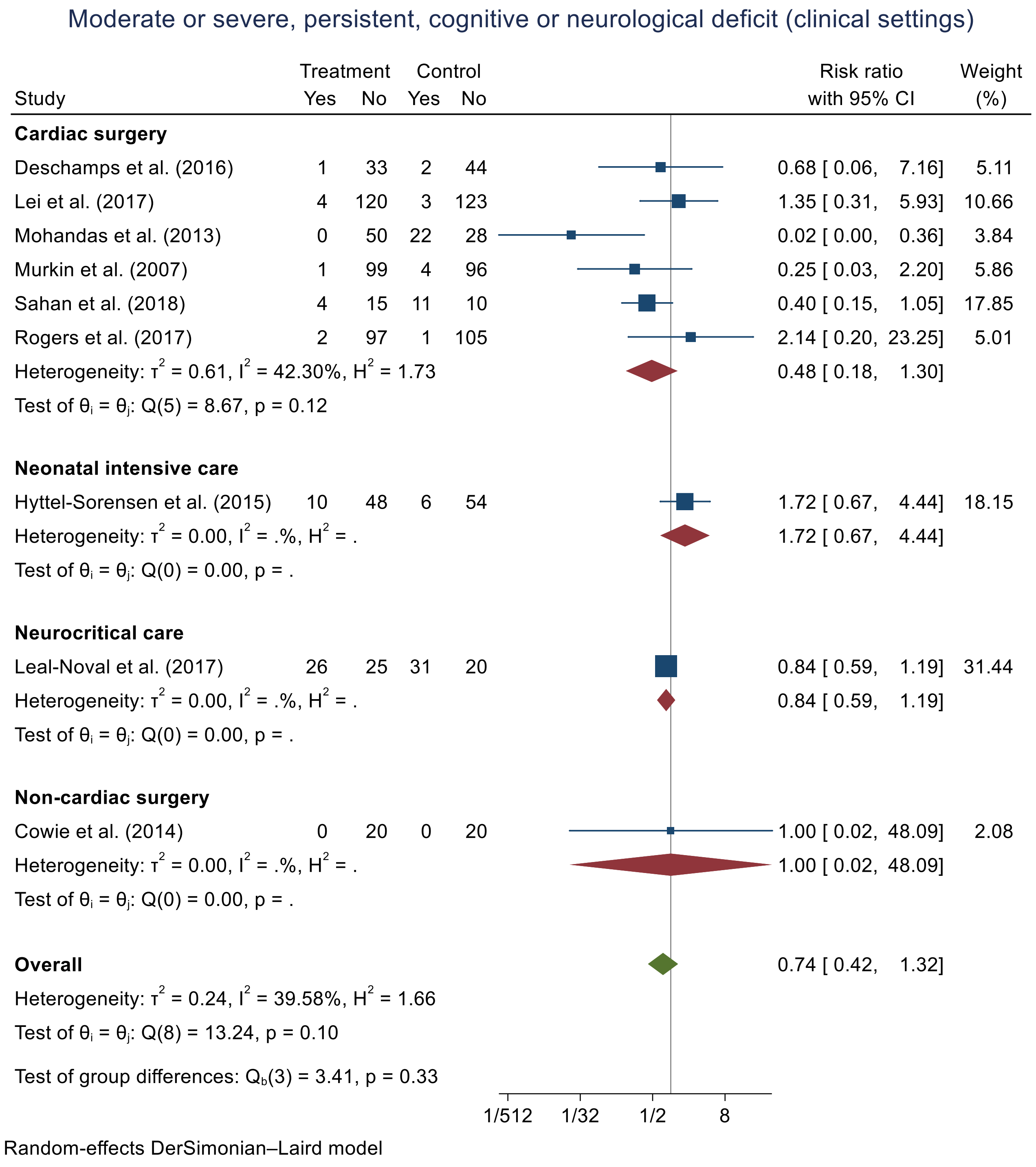

Industry funding (funding not reported compared to industry funded compared to no industry funding)

Figure S14

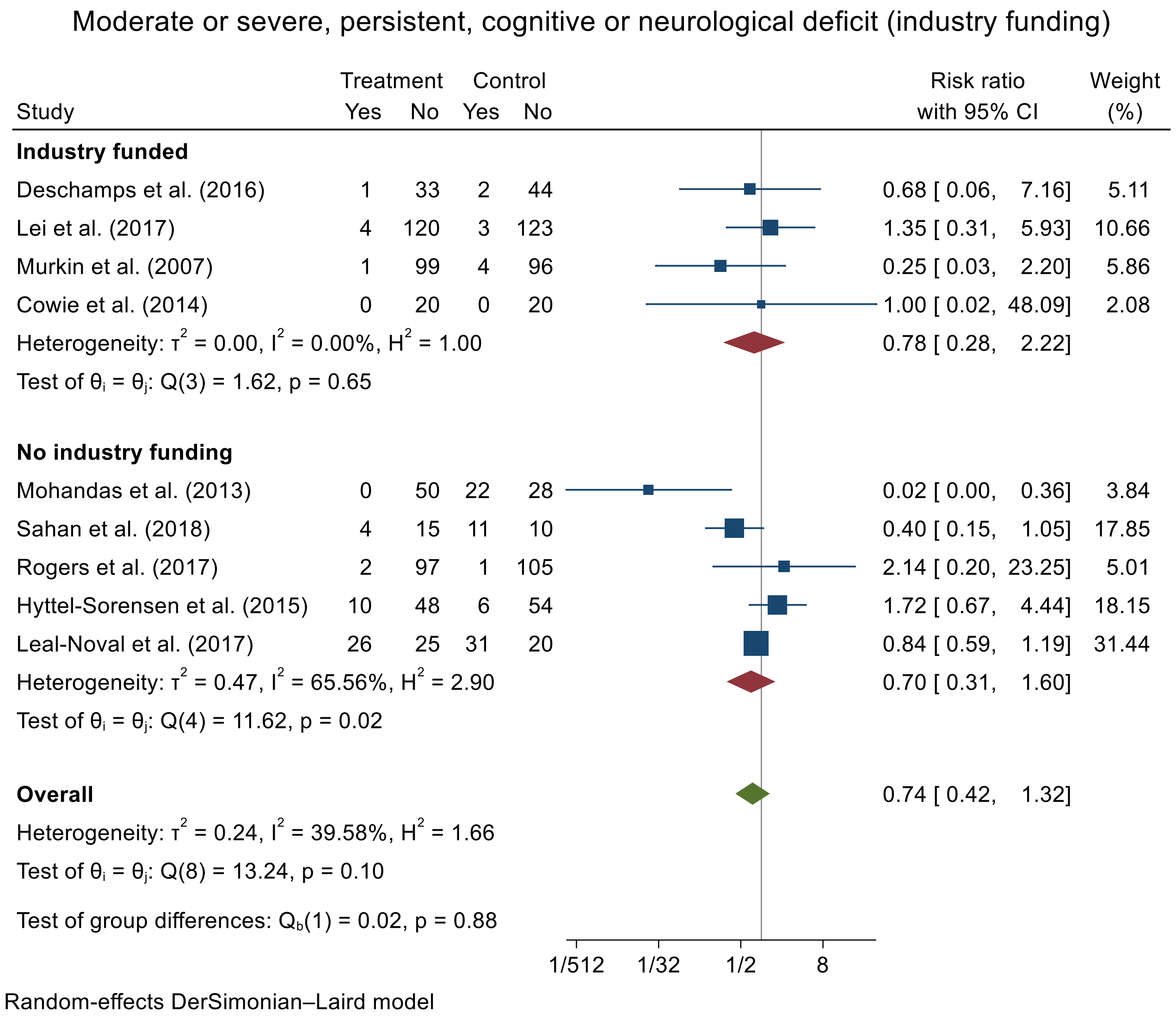

Cerebral NIRS monitoring in the control group (blinded cerebral NIRS monitoring in the control group compared to no cerebral NIRS monitoring in the control group)
Figure S15

Moderate or severe, persistent, cognitive or neurological deficit (blinded cerebral NIRS monitoring)

Study	Treatment Yes	Treatment No	Control Yes	Control No	Risk ratio with 95% CI	Weight (%)
**Blinded cerebral NIRS monitoring in control group**						
Deschamps et al. (2016)	1	33	2	44	0.68 [0.06, 7.16]	5.11
Lei et al. (2017)	4	120	3	123	1.35 [0.31, 5.93]	10.66
Mohandas et al. (2013)	0	50	22	28	0.02 [0.00, 0.36]	3.84
Murkin et al. (2007)	1	99	4	96	0.25 [0.03, 2.20]	5.86
Rogers et al. (2017)	2	97	1	105	2.14 [0.20, 23.25]	5.01
Cowie et al. (2014)	0	20	0	20	1.00 [0.02, 48.09]	2.08
Hyttel-Sorensen et al. (2015)	10	48	6	54	1.72 [0.67, 4.44]	18.15
Heterogeneity: $\tau^2 = 0.74$, $I^2 = 44.04\%$, $H^2 = 1.79$					0.74 [0.27, 2.03]	
Test of $\theta_i = \theta_j$: Q(6) = 10.72, p = 0.10						
**No cerebral NIRS monitoring in control group**						
Sahan et al. (2018)	4	15	11	10	0.40 [0.15, 1.05]	17.85
Leal-Noval et al. (2017)	26	25	31	20	0.84 [0.59, 1.19]	31.44
Heterogeneity: $\tau^2 = 0.13$, $I^2 = 49.71\%$, $H^2 = 1.99$					0.67 [0.34, 1.30]	
Test of $\theta_i = \theta_j$: Q(1) = 1.99, p = 0.16						
**Overall**					0.74 [0.42, 1.32]	
Heterogeneity: $\tau^2 = 0.24$, $I^2 = 39.58\%$, $H^2 = 1.66$						
Test of $\theta_i = \theta_j$: Q(8) = 13.24, p = 0.10						
Test of group differences: $Q_b(1) = 0.03$, p = 0.87						

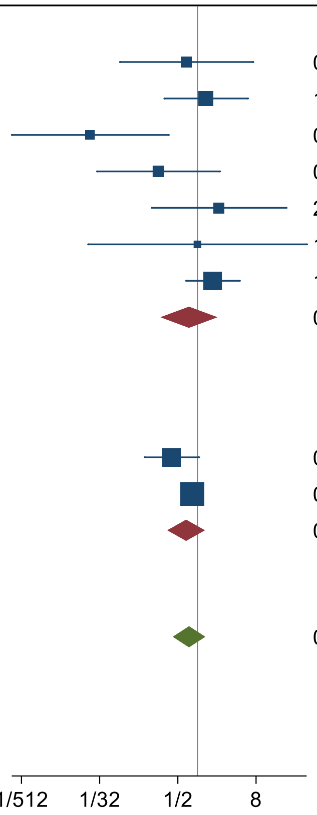

Random-effects DerSimonian–Laird model

Trial Sequential Analysis

Figure S16 – TSA moderate or severe, persistent, cognitive or neurologic deficit

Boundary DARIS Pc 13.8; RR 20%; alpha 2.5%; beta 10%; diversity 78.9 is ignored due to too little information size (3.4% out of 33679).

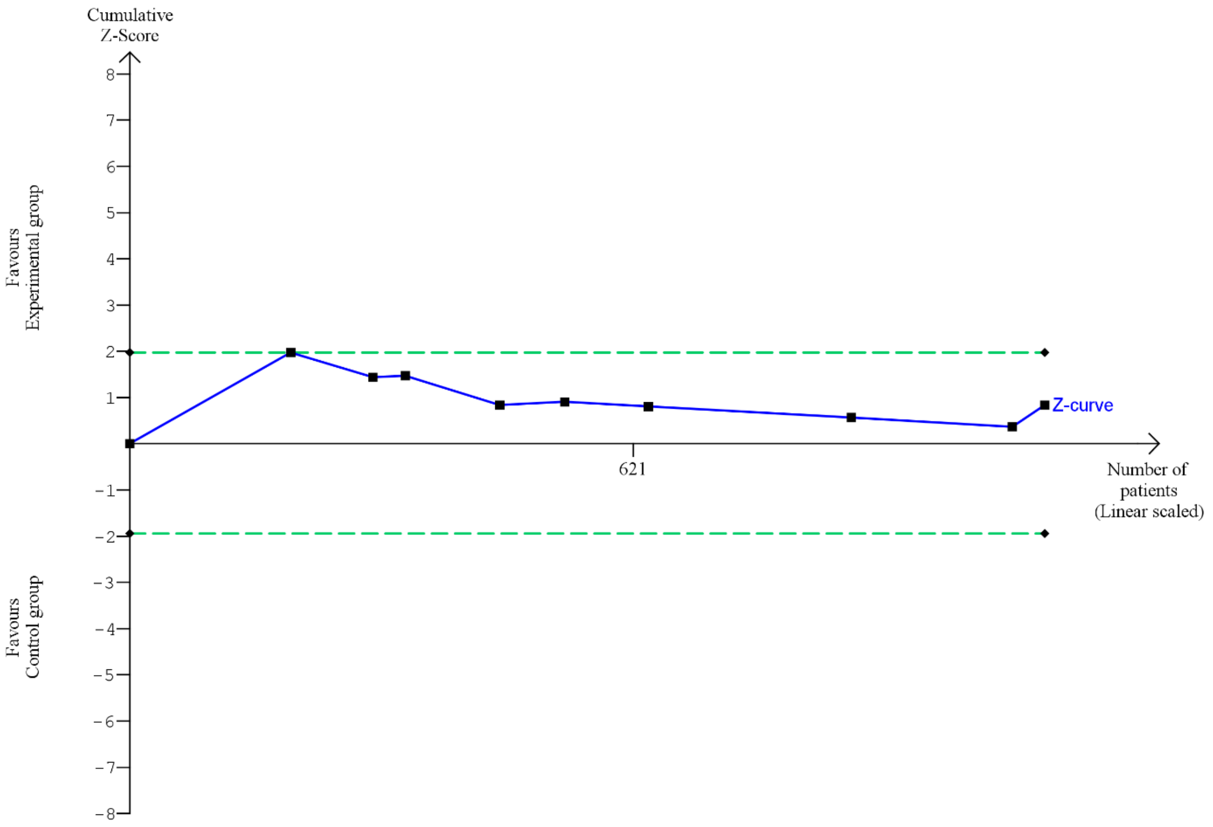

Bayes factor

LnRR meta-analysis = -0,30
LnSE meta-analysis = 0.29
LnRR a priori = -0.22

Bayes factor = 0.60

Sensitivity analyses

Best-worst case analysis

Assuming a beneficial event in the experimental group and harmful in the control group

Figure S17

Moderate or severe, persistent, cognitive or neurological deficit (best-worst case analysis)

Study	Treatment Yes	Treatment No	Control Yes	Control No	Risk ratio with 95% CI	Weight (%)
Deschamps et al. (2016)	1	33	2	44	0.68 [0.06, 7.16]	4.91
Lei et al. (2017)	4	120	3	123	1.35 [0.31, 5.93]	10.16
Mohandas et al. (2013)	0	50	22	28	0.02 [0.00, 0.36]	3.70
Murkin et al. (2007)	1	99	4	96	0.25 [0.03, 2.20]	5.63
Sahan et al. (2018)	4	17	15	10	0.32 [0.12, 0.81]	17.22
Rogers et al. (2017)	2	199	1	105	1.05 [0.10, 11.50]	4.80
Cowie et al. (2014)	0	20	0	20	1.00 [0.02, 48.09]	2.01
Hyttel-Sorensen et al. (2015)	10	76	26	54	0.36 [0.18, 0.69]	22.55
Leal-Noval et al. (2017)	26	25	31	20	0.84 [0.59, 1.19]	29.02
**Overall**					0.50 [0.29, 0.89]	

Heterogeneity: $\tau^2 = 0.26$, $I^2 = 46.62\%$, $H^2 = 1.87$

Test of $\theta_i = \theta_j$: Q(8) = 14.99, p = 0.06

Test of $\theta = 0$: z = -2.37, p = 0.02

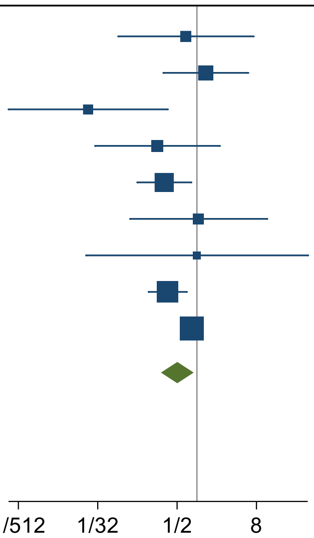

Random-effects DerSimonian–Laird model

Worst-best case analysis

Assuming a harmful event in the experimental group and beneficial in the control group

Figure S18

Moderate or severe, persistent, cognitive or neurologic deficit (worst-best case analysis)

Study	Treatment Yes	Treatment No	Control Yes	Control No	Risk ratio with 95% CI	Weight (%)
Deschamps et al. (2016)	1	33	2	44	0.68 [0.06, 7.16]	7.72
Lei et al. (2017)	4	120	3	123	1.35 [0.31, 5.93]	12.29
Mohandas et al. (2013)	0	50	22	28	0.02 [0.00, 0.36]	6.25
Murkin et al. (2007)	1	99	4	96	0.25 [0.03, 2.20]	8.51
Sahan et al. (2018)	6	15	11	14	0.65 [0.29, 1.46]	16.76
Rogers et al. (2017)	5	97	1	105	5.20 [0.62, 43.71]	8.71
Cowie et al. (2014)	0	20	0	20	1.00 [0.02, 48.09]	3.79
Hyttel-Sorensen et al. (2015)	38	48	6	74	5.89 [2.63, 13.18]	16.78
Leal-Noval et al. (2017)	26	25	31	20	0.84 [0.59, 1.19]	19.19
**Overall**					0.99 [0.43, 2.28]	

Heterogeneity: $\tau^2 = 0.92$, $I^2 = 75.03\%$, $H^2 = 4.01$

Test of $\theta_i = \theta_j$: Q(8) = 32.04, p = 0.00

Test of $\theta = 0$: z = -0.03, p = 0.97

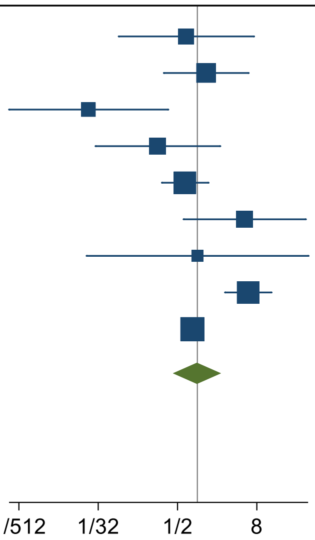

Random-effects DerSimonian–Laird model

Heterogeneity (excluding Mohandas et al 2013)

Figure S19

Moderate or severe, persistent, cognitive or neurologic deficit (excluding Mohandas et al 2013)

Study	Treatment Yes	Treatment No	Control Yes	Control No	Risk ratio with 95% CI	Weight (%)
Deschamps et al. (2016)	1	33	2	44	0.68 [0.06, 7.16]	1.55
Lei et al. (2017)	4	120	3	123	1.35 [0.31, 5.93]	3.97
Murkin et al. (2007)	1	99	4	96	0.25 [0.03, 2.20]	1.83
Sahan et al. (2018)	4	15	11	10	0.40 [0.15, 1.05]	9.36
Rogers et al. (2017)	2	97	1	105	2.14 [0.20, 23.25]	1.52
Cowie et al. (2014)	0	20	0	20	1.00 [0.02, 48.09]	0.58
Hyttel-Sorensen et al. (2015)	10	48	6	54	1.72 [0.67, 4.44]	9.68
Leal-Noval et al. (2017)	26	25	31	20	0.84 [0.59, 1.19]	71.51
**Overall**					0.85 [0.63, 1.14]	

Heterogeneity: $\tau^2 = 0.00$, $I^2 = 0.00\%$, $H^2 = 1.00$

Test of $\theta_i = \theta_j$: $Q(7) = 6.71$, $p = 0.46$

Test of $\theta = 0$: $z = -1.11$, $p = 0.27$

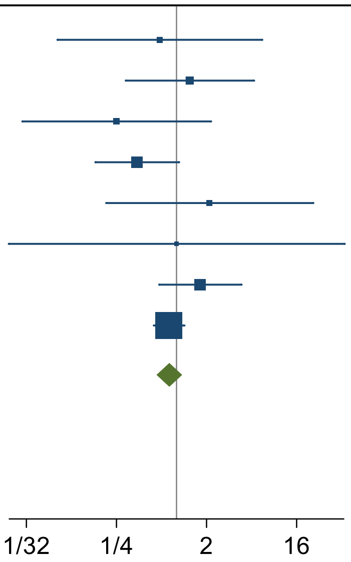

Random-effects DerSimonian–Laird model

Proportion of participants with one or more serious adverse events

Primary analysis

Figure S20 – fixed effect

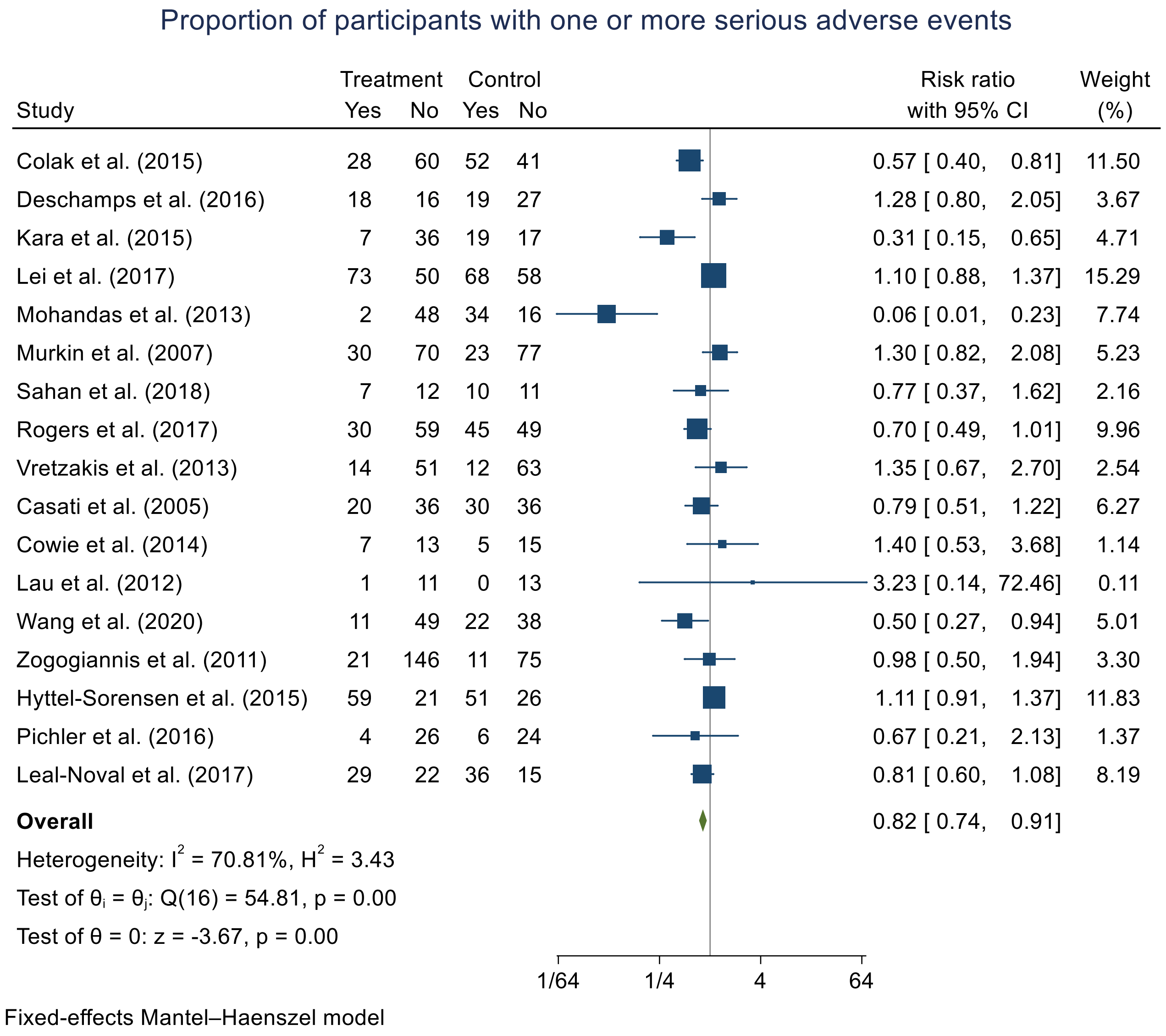

Figure S21 – random effects

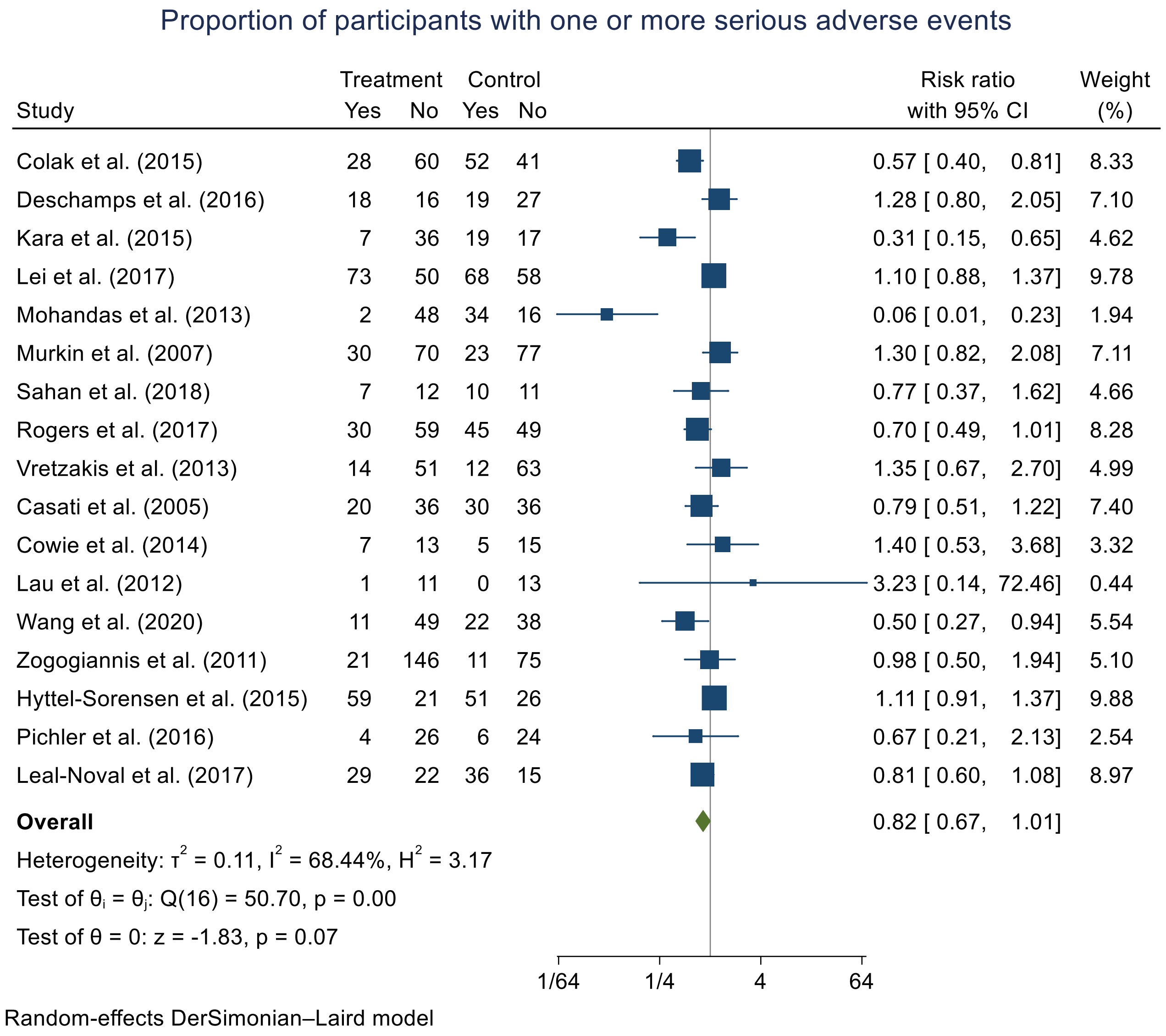

Subgroup analyses

Risk of bias (high compared to low)

Figure S22

Proportion of participants with one or more serious adverse events (risk of bias)

Study	Treatment Yes	Treatment No	Control Yes	Control No	Risk ratio with 95% CI	Weight (%)
**High risk of bias**						
Colak et al. (2015)	28	60	52	41	0.57 [0.40, 0.81]	8.33
Deschamps et al. (2016)	18	16	19	27	1.28 [0.80, 2.05]	7.10
Kara et al. (2015)	7	36	19	17	0.31 [0.15, 0.65]	4.62
Lei et al. (2017)	73	50	68	58	1.10 [0.88, 1.37]	9.78
Mohandas et al. (2013)	2	48	34	16	0.06 [0.01, 0.23]	1.94
Murkin et al. (2007)	30	70	23	77	1.30 [0.82, 2.08]	7.11
Sahan et al. (2018)	7	12	10	11	0.77 [0.37, 1.62]	4.66
Rogers et al. (2017)	30	59	45	49	0.70 [0.49, 1.01]	8.28
Vretzakis et al. (2013)	14	51	12	63	1.35 [0.67, 2.70]	4.99
Casati et al. (2005)	20	36	30	36	0.79 [0.51, 1.22]	7.40
Cowie et al. (2014)	7	13	5	15	1.40 [0.53, 3.68]	3.32
Lau et al. (2012)	1	11	0	13	3.23 [0.14, 72.46]	0.44
Wang et al. (2020)	11	49	22	38	0.50 [0.27, 0.94]	5.54
Zogogiannis et al. (2011)	21	146	11	75	0.98 [0.50, 1.94]	5.10
Pichler et al. (2016)	4	26	6	24	0.67 [0.21, 2.13]	2.54
Leal-Noval et al. (2017)	29	22	36	15	0.81 [0.60, 1.08]	8.97
Heterogeneity: $\tau^2 = 0.12$, $I^2 = 67.38\%$, $H^2 = 3.07$					0.79 [0.63, 1.00]	
Test of $\theta_i = \theta_j$: Q(15) = 45.98, p = 0.00						
**Low risk of bias**						
Hyttel-Sorensen et al. (2015)	59	21	51	26	1.11 [0.91, 1.37]	9.88
Heterogeneity: $\tau^2 = 0.00$, $I^2 = .\%$, $H^2 = .$					1.11 [0.91, 1.37]	
Test of $\theta_i = \theta_j$: Q(0) = 0.00, p = .						
**Overall**					0.82 [0.67, 1.01]	
Heterogeneity: $\tau^2 = 0.11$, $I^2 = 68.44\%$, $H^2 = 3.17$						
Test of $\theta_i = \theta_j$: Q(16) = 50.70, p = 0.00						
Test of group differences: $Q_b(1) = 4.63$, p = 0.03						

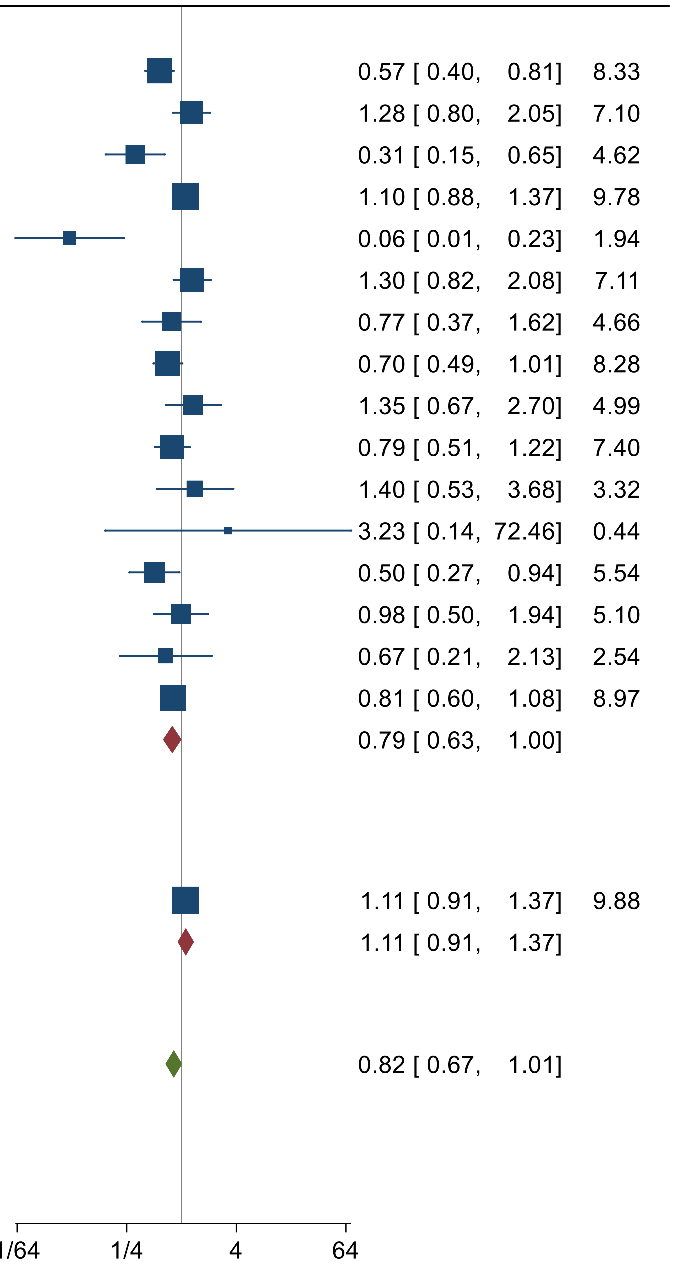

Random-effects DerSimonian–Laird model

Clinical setting (cardiac surgery compared to neonatal intensive care compared to neurocritical care compared to non-cardiac surgery)
Figure S23

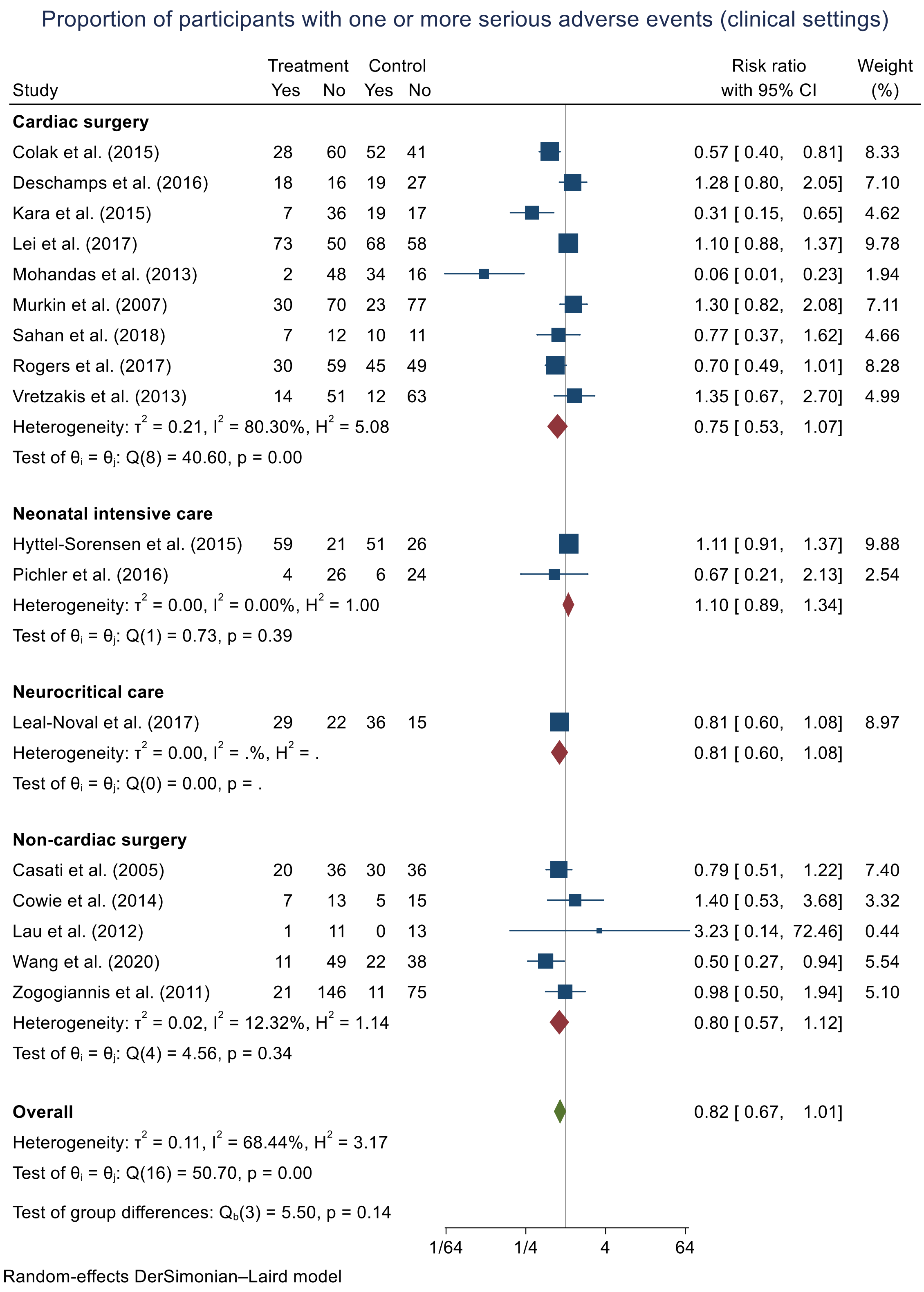

Industry funding (funding not reported compared to industry funded compared to no industry funding)
Figure S24

Proportion of participants with one or more serious adverse events (industry funding)

Study	Treatment Yes	Treatment No	Control Yes	Control No	Risk ratio with 95% CI	Weight (%)
**Funding not reported**						
Lau et al. (2012)	1	11	0	13	3.23 [0.14, 72.46]	0.44
Heterogeneity: τ^2 = 0.00, I^2 = .%, H^2 = .					3.23 [0.14, 72.46]	
Test of $\theta_i = \theta_j$: Q(0) = -0.00, p = .						
**Industry funded**						
Deschamps et al. (2016)	18	16	19	27	1.28 [0.80, 2.05]	7.10
Lei et al. (2017)	73	50	68	58	1.10 [0.88, 1.37]	9.78
Murkin et al. (2007)	30	70	23	77	1.30 [0.82, 2.08]	7.11
Casati et al. (2005)	20	36	30	36	0.79 [0.51, 1.22]	7.40
Cowie et al. (2014)	7	13	5	15	1.40 [0.53, 3.68]	3.32
Heterogeneity: τ^2 = 0.00, I^2 = 0.00%, H^2 = 1.00					1.10 [0.93, 1.30]	
Test of $\theta_i = \theta_j$: Q(4) = 3.41, p = 0.49						
**No industry funding**						
Colak et al. (2015)	28	60	52	41	0.57 [0.40, 0.81]	8.33
Kara et al. (2015)	7	36	19	17	0.31 [0.15, 0.65]	4.62
Mohandas et al. (2013)	2	48	34	16	0.06 [0.01, 0.23]	1.94
Sahan et al. (2018)	7	12	10	11	0.77 [0.37, 1.62]	4.66
Rogers et al. (2017)	30	59	45	49	0.70 [0.49, 1.01]	8.28
Vretzakis et al. (2013)	14	51	12	63	1.35 [0.67, 2.70]	4.99
Wang et al. (2020)	11	49	22	38	0.50 [0.27, 0.94]	5.54
Zogogiannis et al. (2011)	21	146	11	75	0.98 [0.50, 1.94]	5.10
Hyttel-Sorensen et al. (2015)	59	21	51	26	1.11 [0.91, 1.37]	9.88
Pichler et al. (2016)	4	26	6	24	0.67 [0.21, 2.13]	2.54
Leal-Noval et al. (2017)	29	22	36	15	0.81 [0.60, 1.08]	8.97
Heterogeneity: τ^2 = 0.15, I^2 = 74.20%, H^2 = 3.88					0.68 [0.50, 0.91]	
Test of $\theta_i = \theta_j$: Q(10) = 38.76, p = 0.00						
**Overall**					0.82 [0.67, 1.01]	
Heterogeneity: τ^2 = 0.11, I^2 = 68.44%, H^2 = 3.17						
Test of $\theta_i = \theta_j$: Q(16) = 50.70, p = 0.00						
Test of group differences: $Q_b(2)$ = 8.50, p = 0.01						

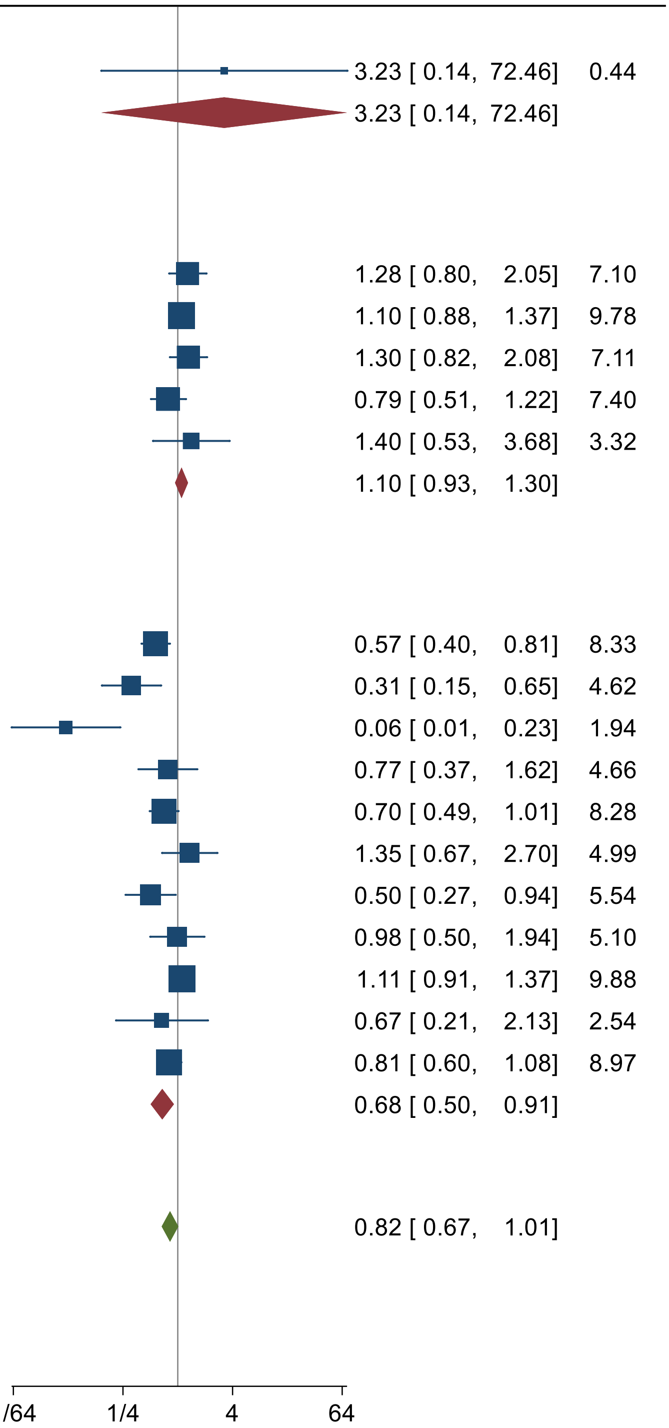

Random-effects DerSimonian–Laird model

Cerebral NIRS monitoring in the control group (blinded cerebral NIRS monitoring in the control group compared to no cerebral NIRS monitoring in the control group)

Figure S25

Proportion of participants with one or more serious adverse events (blinded cerebral NIRS monitoring)

Study	Treatment Yes	Treatment No	Control Yes	Control No	Risk ratio with 95% CI	Weight (%)
**Blinded cerebral NIRS monitoring in control group**						
Deschamps et al. (2016)	18	16	19	27	1.28 [0.80, 2.05]	7.10
Lei et al. (2017)	73	50	68	58	1.10 [0.88, 1.37]	9.78
Mohandas et al. (2013)	2	48	34	16	0.06 [0.01, 0.23]	1.94
Murkin et al. (2007)	30	70	23	77	1.30 [0.82, 2.08]	7.11
Rogers et al. (2017)	30	59	45	49	0.70 [0.49, 1.01]	8.28
Casati et al. (2005)	20	36	30	36	0.79 [0.51, 1.22]	7.40
Cowie et al. (2014)	7	13	5	15	1.40 [0.53, 3.68]	3.32
Lau et al. (2012)	1	11	0	13	3.23 [0.14, 72.46]	0.44
Wang et al. (2020)	11	49	22	38	0.50 [0.27, 0.94]	5.54
Hyttel-Sorensen et al. (2015)	59	21	51	26	1.11 [0.91, 1.37]	9.88
Pichler et al. (2016)	4	26	6	24	0.67 [0.21, 2.13]	2.54
Heterogeneity: $\tau^2 = 0.10$, $I^2 = 68.21\%$, $H^2 = 3.15$					0.89 [0.68, 1.15]	
Test of $\theta_i = \theta_j$: Q(10) = 31.45, p = 0.00						
**No cerebral NIRS monitoring in control group**						
Colak et al. (2015)	28	60	52	41	0.57 [0.40, 0.81]	8.33
Kara et al. (2015)	7	36	19	17	0.31 [0.15, 0.65]	4.62
Sahan et al. (2018)	7	12	10	11	0.77 [0.37, 1.62]	4.66
Vretzakis et al. (2013)	14	51	12	63	1.35 [0.67, 2.70]	4.99
Zogogiannis et al. (2011)	21	146	11	75	0.98 [0.50, 1.94]	5.10
Leal-Noval et al. (2017)	29	22	36	15	0.81 [0.60, 1.08]	8.97
Heterogeneity: $\tau^2 = 0.08$, $I^2 = 55.17\%$, $H^2 = 2.23$					0.72 [0.52, 1.00]	
Test of $\theta_i = \theta_j$: Q(5) = 11.15, p = 0.05						
**Overall**					0.82 [0.67, 1.01]	
Heterogeneity: $\tau^2 = 0.11$, $I^2 = 68.44\%$, $H^2 = 3.17$						
Test of $\theta_i = \theta_j$: Q(16) = 50.70, p = 0.00						
Test of group differences: $Q_b(1) = 0.94$, p = 0.33						

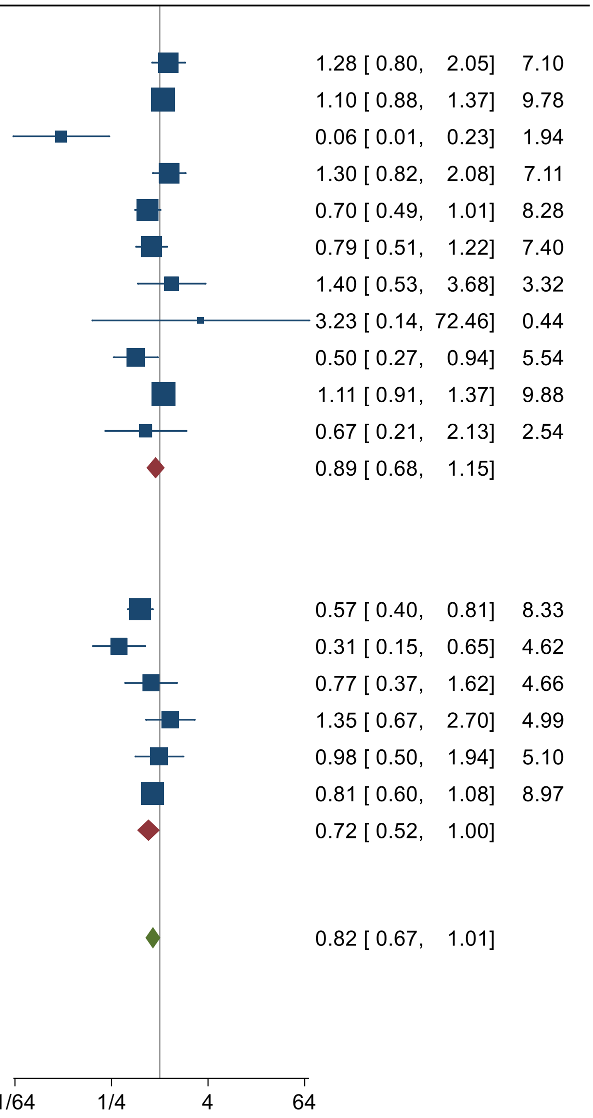

Random-effects DerSimonian–Laird model

Trial Sequential Analysis

Figure S26 – TSA proportion of participants with one or more serious adverse events

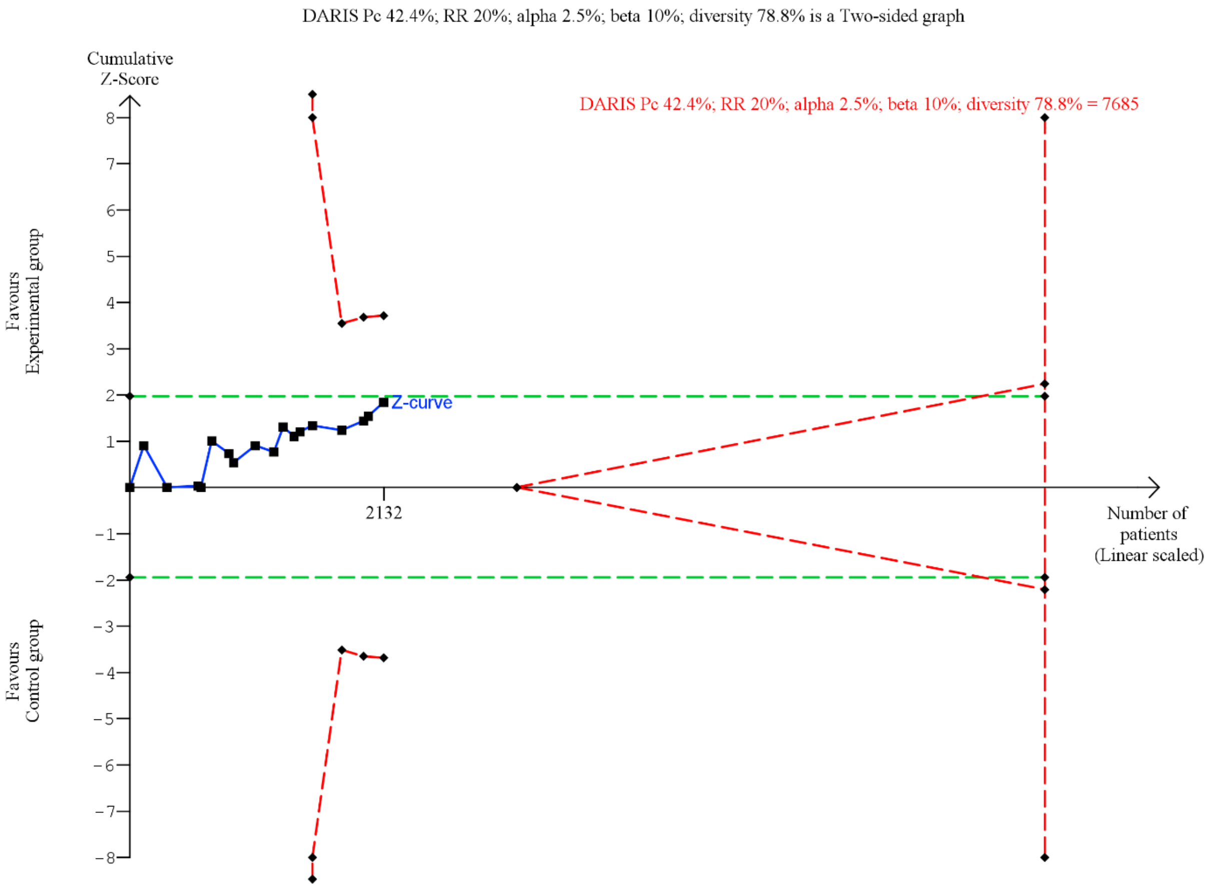

Trial Sequential analysis adjusted CI: 0.56 to 1.20 (p=0.07)

Bayes factor

LnRR meta-analysis = -0.20
LnSE meta-analysis = 0.10
LnRR a priori = -0.22

Bayes factor = 0.14

Sensitivity analyses

Best-worst case analysis

Assuming a beneficial event in the experimental group and harmful in the control group

Figure S27

Proportion of participants with one or more serious adverse events (best-worst case analysis

Study	Treatment Yes	Treatment No	Control Yes	Control No	Risk ratio with 95% CI	Weight (%)
Colak et al. (2015)	28	72	59	41	0.47 [0.33, 0.68]	8.08
Deschamps et al. (2016)	18	16	19	27	1.28 [0.80, 2.05]	7.06
Kara et al. (2015)	7	36	19	17	0.31 [0.15, 0.65]	4.87
Lei et al. (2017)	73	51	68	58	1.09 [0.88, 1.36]	9.17
Mohandas et al. (2013)	2	48	34	16	0.06 [0.01, 0.23]	2.19
Murkin et al. (2007)	30	70	23	77	1.30 [0.82, 2.08]	7.07
Sahan et al. (2018)	7	14	14	11	0.60 [0.30, 1.20]	5.19
Rogers et al. (2017)	30	72	57	49	0.55 [0.39, 0.78]	8.12
Vretzakis et al. (2013)	14	51	12	63	1.35 [0.67, 2.70]	5.21
Casati et al. (2005)	20	43	32	36	0.67 [0.43, 1.05]	7.30
Cowie et al. (2014)	7	13	5	15	1.40 [0.53, 3.68]	3.61
Lau et al. (2012)	1	11	0	13	3.23 [0.14, 72.46]	0.52
Wang et al. (2020)	11	49	22	38	0.50 [0.27, 0.94]	5.71
Zogogiannis et al. (2011)	21	146	11	75	0.98 [0.50, 1.94]	5.31
Hyttel-Sorensen et al. (2015)	59	27	54	26	1.02 [0.82, 1.25]	9.24
Pichler et al. (2016)	4	26	6	24	0.67 [0.21, 2.13]	2.81
Leal-Noval et al. (2017)	29	22	36	15	0.81 [0.60, 1.08]	8.56
**Overall**					0.76 [0.60, 0.96]	

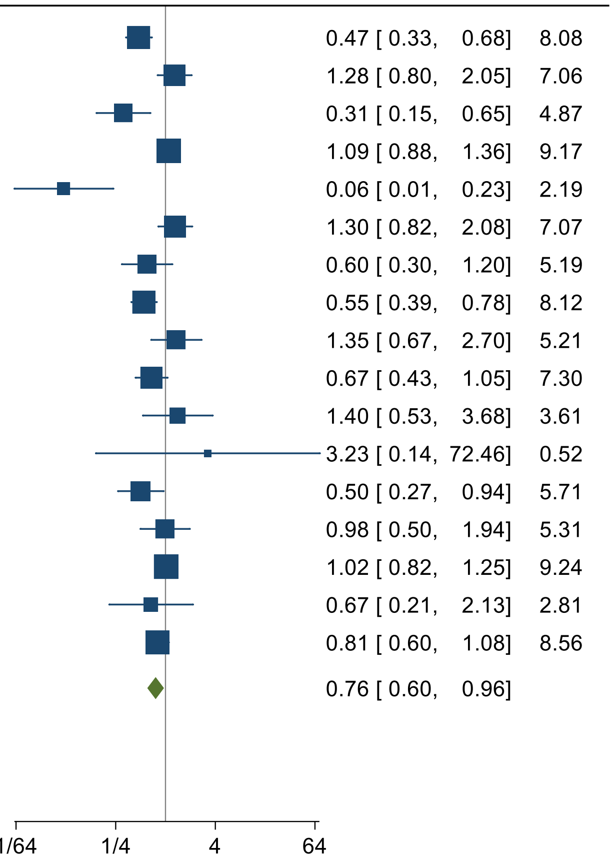

Heterogeneity: $\tau^2 = 0.14$, $I^2 = 73.73\%$, $H^2 = 3.81$

Test of $\theta_i = \theta_j$: Q(16) = 60.91, p = 0.00

Test of $\theta = 0$: z = -2.35, p = 0.02

Random-effects DerSimonian–Laird model

Worst-best case analysis

Assuming a harmful event in the experimental group and beneficial in the control group

Figure S28

Proportion of participants with one or more serious adverse events (worst-best case analysis)

Study	Treatment Yes	Treatment No	Control Yes	Control No	Risk ratio with 95% CI	Weight (%)
Colak et al. (2015)	40	60	52	48	0.77 [0.57, 1.04]	9.12
Deschamps et al. (2016)	18	16	19	27	1.28 [0.80, 2.05]	6.89
Kara et al. (2015)	7	36	19	17	0.31 [0.15, 0.65]	4.17
Lei et al. (2017)	73	51	68	58	1.09 [0.88, 1.36]	10.32
Mohandas et al. (2013)	2	48	34	16	0.06 [0.01, 0.23]	1.63
Murkin et al. (2007)	30	70	23	77	1.30 [0.82, 2.08]	6.90
Sahan et al. (2018)	9	12	10	15	1.07 [0.54, 2.13]	4.61
Rogers et al. (2017)	43	59	45	61	0.99 [0.72, 1.36]	8.94
Vretzakis et al. (2013)	14	51	12	63	1.35 [0.67, 2.70]	4.55
Casati et al. (2005)	27	36	30	38	0.97 [0.66, 1.44]	7.91
Cowie et al. (2014)	7	13	5	15	1.40 [0.53, 3.68]	2.89
Lau et al. (2012)	1	11	0	13	3.23 [0.14, 72.46]	0.36
Wang et al. (2020)	11	49	22	38	0.50 [0.27, 0.94]	5.13
Zogogiannis et al. (2011)	21	146	11	75	0.98 [0.50, 1.94]	4.67
Hyttel-Sorensen et al. (2015)	65	21	51	29	1.19 [0.97, 1.45]	10.50
Pichler et al. (2016)	4	26	6	24	0.67 [0.21, 2.13]	2.17
Leal-Noval et al. (2017)	29	22	36	15	0.81 [0.60, 1.08]	9.23
**Overall**					0.91 [0.75, 1.10]	

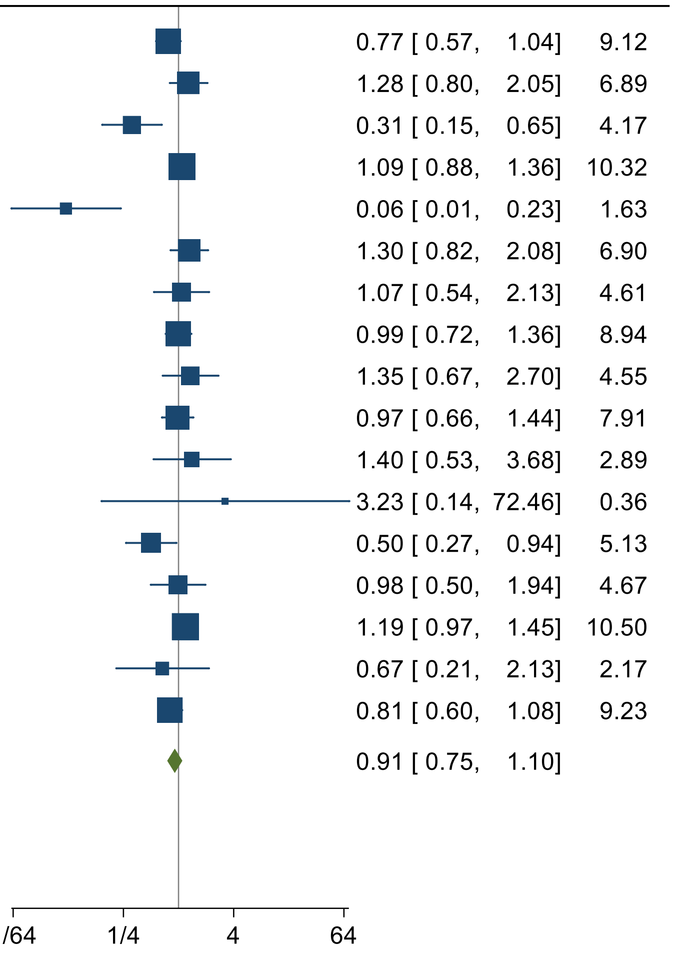

Heterogeneity: $\tau^2 = 0.08$, $I^2 = 63.00\%$, $H^2 = 2.70$

Test of $\theta_i = \theta_j$: $Q(16) = 43.25$, $p = 0.00$

Test of $\theta = 0$: $z = -0.98$, $p = 0.33$

Random-effects DerSimonian–Laird model

Heterogeneity (excluding Mohandas et al. 2013)

Figure S29

Proportion of participants with one or more serious adverse events (excluding Mohandas et al 2013)

Study	Treatment Yes	Treatment No	Control Yes	Control No	Risk ratio with 95% CI	Weight (%)
Colak et al. (2015)	28	60	52	41	0.57 [0.40, 0.81]	8.91
Deschamps et al. (2016)	18	16	19	27	1.28 [0.80, 2.05]	7.11
Kara et al. (2015)	7	36	19	17	0.31 [0.15, 0.65]	4.10
Lei et al. (2017)	73	50	68	58	1.10 [0.88, 1.37]	11.35
Murkin et al. (2007)	30	70	23	77	1.30 [0.82, 2.08]	7.12
Sahan et al. (2018)	7	12	10	11	0.77 [0.37, 1.62]	4.14
Rogers et al. (2017)	30	59	45	49	0.70 [0.49, 1.01]	8.83
Vretzakis et al. (2013)	14	51	12	63	1.35 [0.67, 2.70]	4.50
Casati et al. (2005)	20	36	30	36	0.79 [0.51, 1.22]	7.53
Cowie et al. (2014)	7	13	5	15	1.40 [0.53, 3.68]	2.78
Lau et al. (2012)	1	11	0	13	3.23 [0.14, 72.46]	0.33
Wang et al. (2020)	11	49	22	38	0.50 [0.27, 0.94]	5.13
Zogogiannis et al. (2011)	21	146	11	75	0.98 [0.50, 1.94]	4.63
Hyttel-Sorensen et al. (2015)	59	21	51	26	1.11 [0.91, 1.37]	11.55
Pichler et al. (2016)	4	26	6	24	0.67 [0.21, 2.13]	2.06
Leal-Noval et al. (2017)	29	22	36	15	0.81 [0.60, 1.08]	9.93
**Overall**					0.87 [0.73, 1.04]	

Heterogeneity: $\tau^2 = 0.06$, $I^2 = 57.46\%$, $H^2 = 2.35$

Test of $\theta_i = \theta_j$: $Q(15) = 35.26$, $p = 0.00$

Test of $\theta = 0$: $z = -1.50$, $p = 0.13$

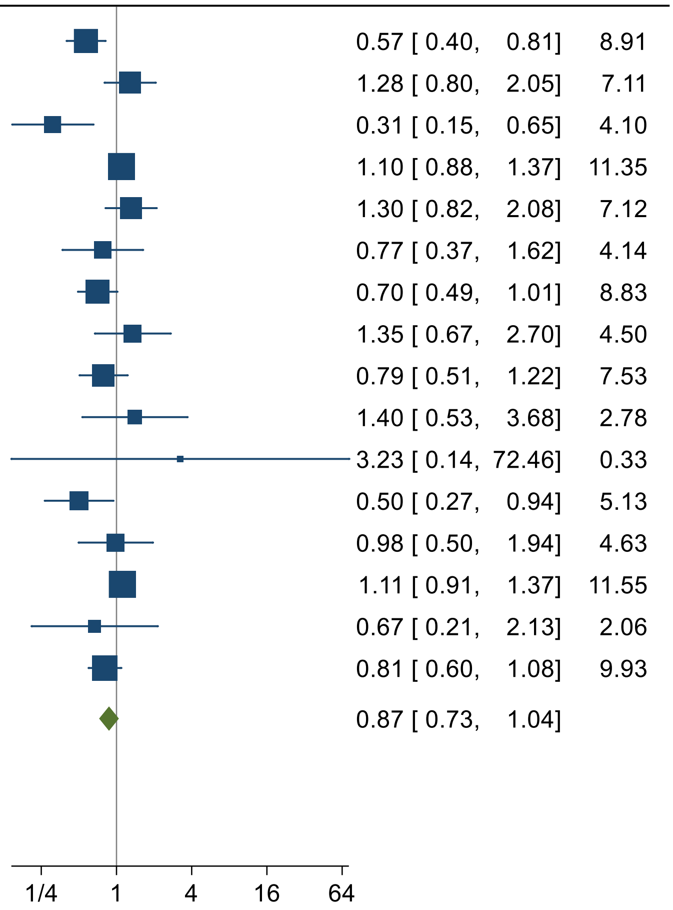

Random-effects DerSimonian–Laird model

Funnel plot

Figure S30

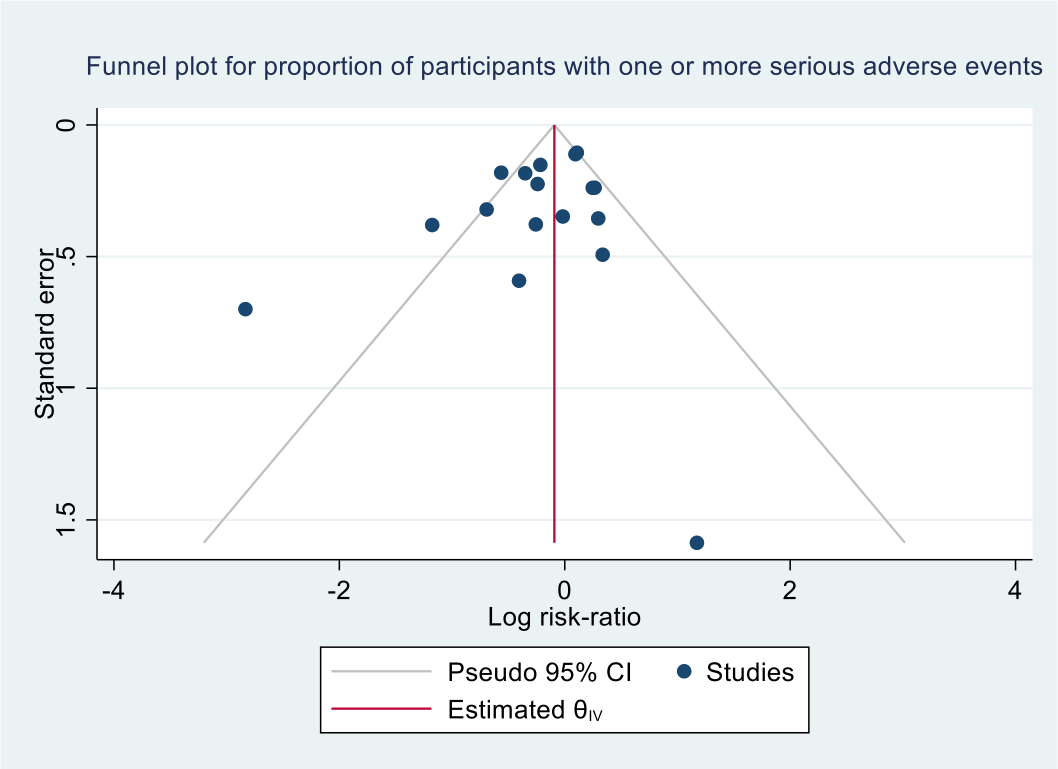

Harbord test: p=0.60

Mild, moderate or severe, temporary or persistent, cognitive or neurological deficit

Primary analysis

Figure S31 – fixed effect

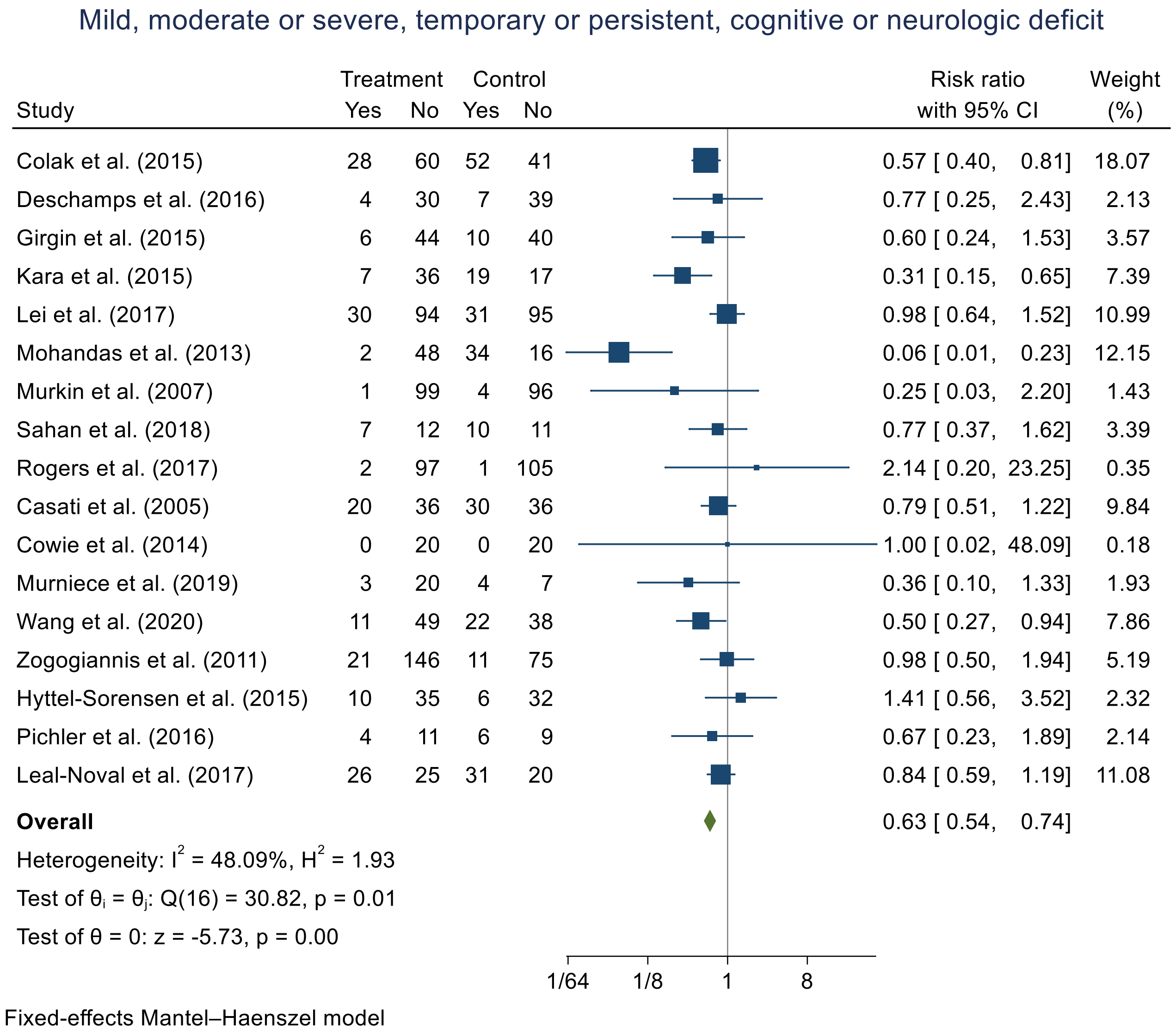

Figure S32 – random effects

Mild, moderate or severe, temporary or persistent, cognitive or neurologic deficit

Study	Treatment Yes	Treatment No	Control Yes	Control No	Risk ratio with 95% CI	Weight (%)
Colak et al. (2015)	28	60	52	41	0.57 [0.40, 0.81]	12.12
Deschamps et al. (2016)	4	30	7	39	0.77 [0.25, 2.43]	3.61
Girgin et al. (2015)	6	44	10	40	0.60 [0.24, 1.53]	4.89
Kara et al. (2015)	7	36	19	17	0.31 [0.15, 0.65]	6.55
Lei et al. (2017)	30	94	31	95	0.98 [0.64, 1.52]	10.75
Mohandas et al. (2013)	2	48	34	16	0.06 [0.01, 0.23]	2.70
Murkin et al. (2007)	1	99	4	96	0.25 [0.03, 2.20]	1.20
Sahan et al. (2018)	7	12	10	11	0.77 [0.37, 1.62]	6.60
Rogers et al. (2017)	2	97	1	105	2.14 [0.20, 23.25]	1.01
Casati et al. (2005)	20	36	30	36	0.79 [0.51, 1.22]	10.70
Cowie et al. (2014)	0	20	0	20	1.00 [0.02, 48.09]	0.40
Murniece et al. (2019)	3	20	4	7	0.36 [0.10, 1.33]	2.90
Wang et al. (2020)	11	49	22	38	0.50 [0.27, 0.94]	7.89
Zogogiannis et al. (2011)	21	146	11	75	0.98 [0.50, 1.94]	7.25
Hyttel-Sorensen et al. (2015)	10	35	6	32	1.41 [0.56, 3.52]	5.02
Pichler et al. (2016)	4	11	6	9	0.67 [0.23, 1.89]	4.16
Leal-Noval et al. (2017)	26	25	31	20	0.84 [0.59, 1.19]	12.25
**Overall**					0.66 [0.51, 0.84]	

Heterogeneity: $\tau^2 = 0.10$, $I^2 = 45.49\%$, $H^2 = 1.83$

Test of $\theta_i = \theta_j$: Q(16) = 29.35, p = 0.02

Test of $\theta = 0$: z = -3.31, p = 0.00

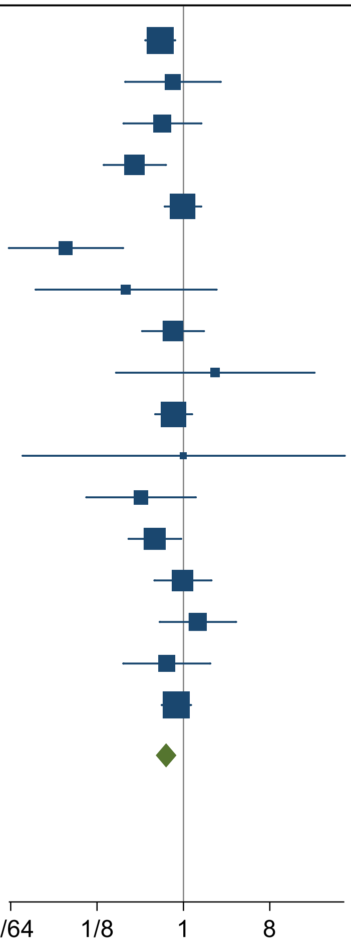

Random-effects DerSimonian–Laird model

Sensitivity analysis

Heterogeneity (excluding Mohandas et al. 2013)

Figure S33

Mild, moderate or severe, temporary or persistent, cognitive or neurologic deficit (excluding Mohandas et al 2013)

Study	Treatment Yes	Treatment No	Control Yes	Control No	Risk ratio with 95% CI	Weight (%)
Colak et al. (2015)	28	60	52	41	0.57 [0.40, 0.81]	17.70
Deschamps et al. (2016)	4	30	7	39	0.77 [0.25, 2.43]	2.28
Girgin et al. (2015)	6	44	10	40	0.60 [0.24, 1.53]	3.37
Kara et al. (2015)	7	36	19	17	0.31 [0.15, 0.65]	5.13
Lei et al. (2017)	30	94	31	95	0.98 [0.64, 1.52]	12.94
Murkin et al. (2007)	1	99	4	96	0.25 [0.03, 2.20]	0.65
Sahan et al. (2018)	7	12	10	11	0.77 [0.37, 1.62]	5.19
Rogers et al. (2017)	2	97	1	105	2.14 [0.20, 23.25]	0.54
Casati et al. (2005)	20	36	30	36	0.79 [0.51, 1.22]	12.79
Cowie et al. (2014)	0	20	0	20	1.00 [0.02, 48.09]	0.21
Murniece et al. (2019)	3	20	4	7	0.36 [0.10, 1.33]	1.75
Wang et al. (2020)	11	49	22	38	0.50 [0.27, 0.94]	6.97
Zogogiannis et al. (2011)	21	146	11	75	0.98 [0.50, 1.94]	6.04
Hyttel-Sorensen et al. (2015)	10	35	6	32	1.41 [0.56, 3.52]	3.49
Pichler et al. (2016)	4	11	6	9	0.67 [0.23, 1.89]	2.72
Leal-Noval et al. (2017)	26	25	31	20	0.84 [0.59, 1.19]	18.23
**Overall**					0.72 [0.60, 0.85]	

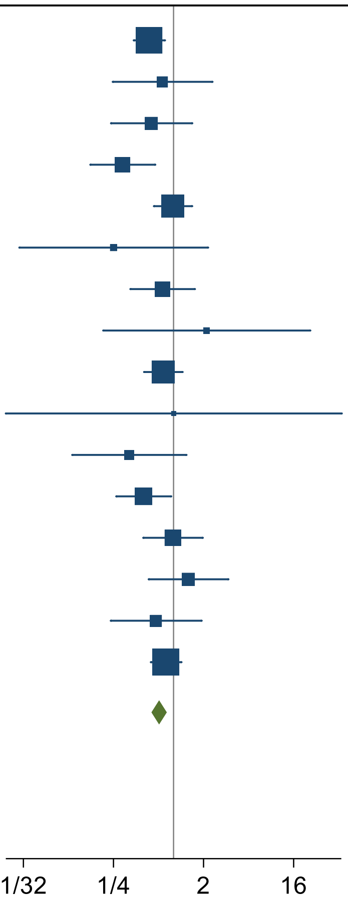

Heterogeneity: $\tau^2 = 0.01$, $I^2 = 10.20\%$, $H^2 = 1.11$

Test of $\theta_i = \theta_j$: Q(15) = 16.70, p = 0.34

Test of θ = 0: z = -3.71, p = 0.00

Random-effects DerSimonian–Laird model

Brain damage on imaging at maximal follow-up

Primary analysis

Figure S34 – fixed effect

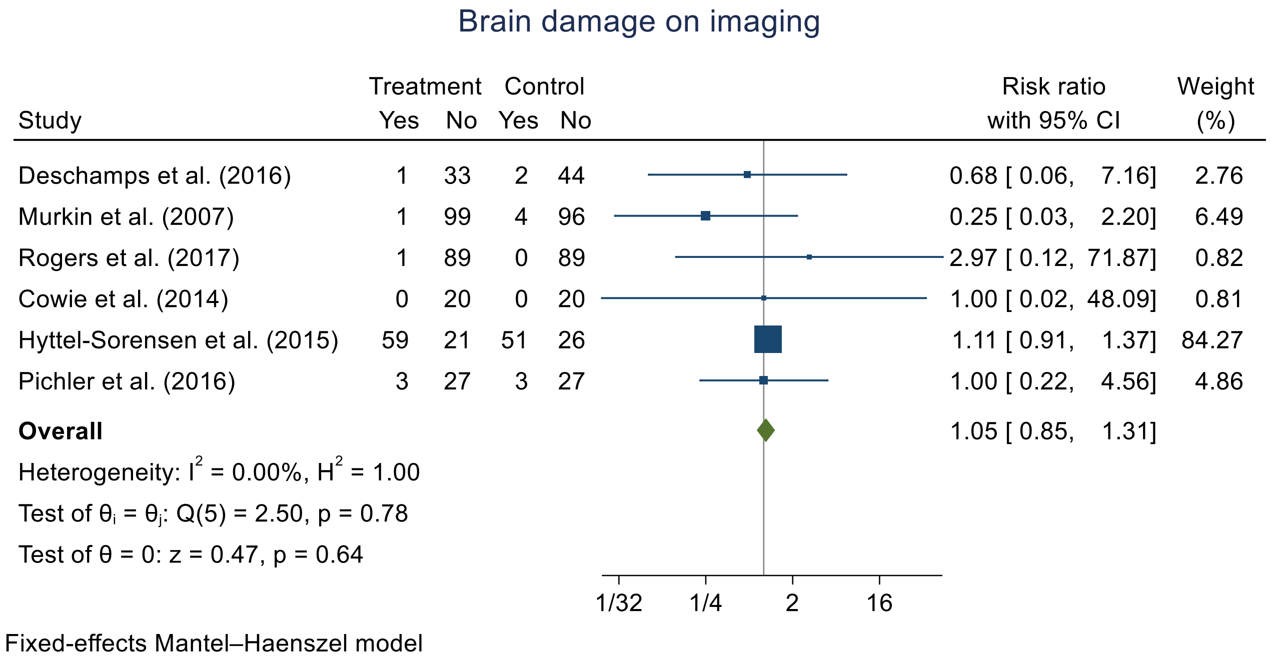

Figure S35 – random effects

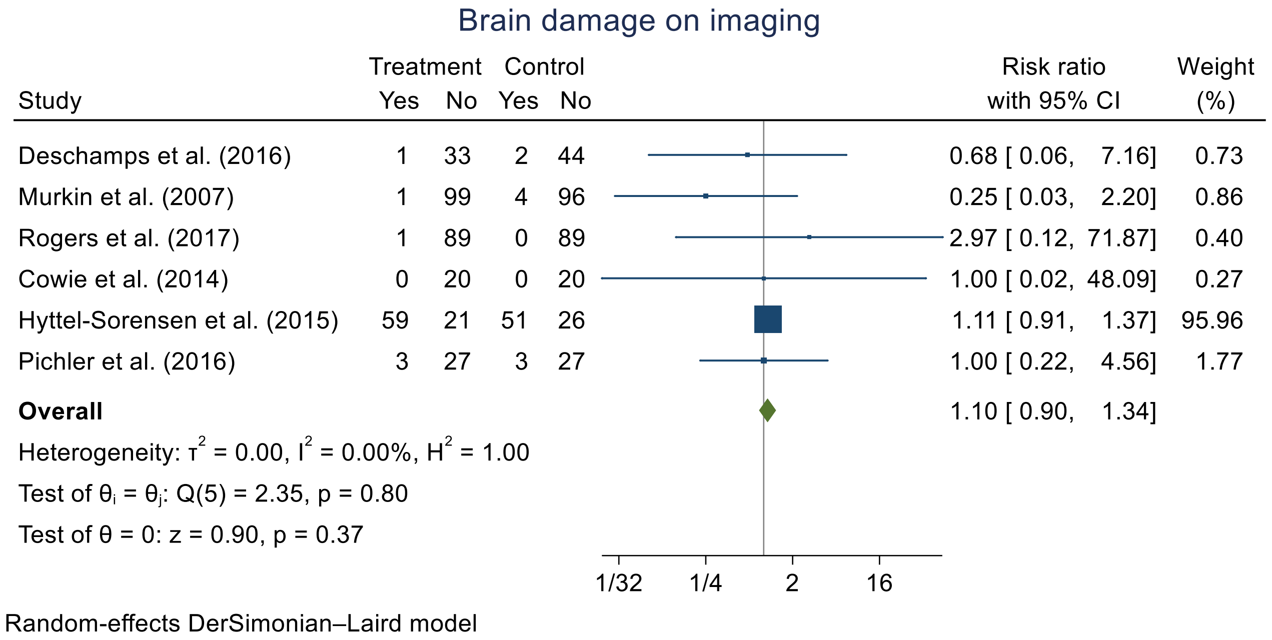

Proportion of participants with one or more adverse events

Primary analysis

Figure S36 – fixed effect

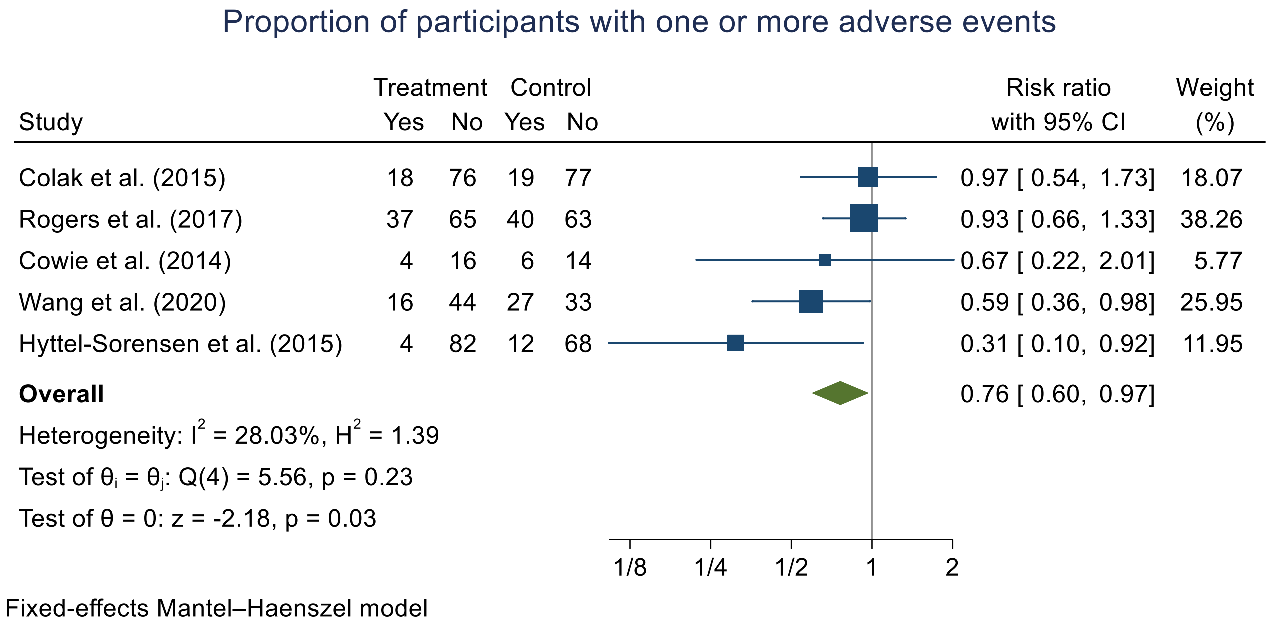

Figure S37 – random effects

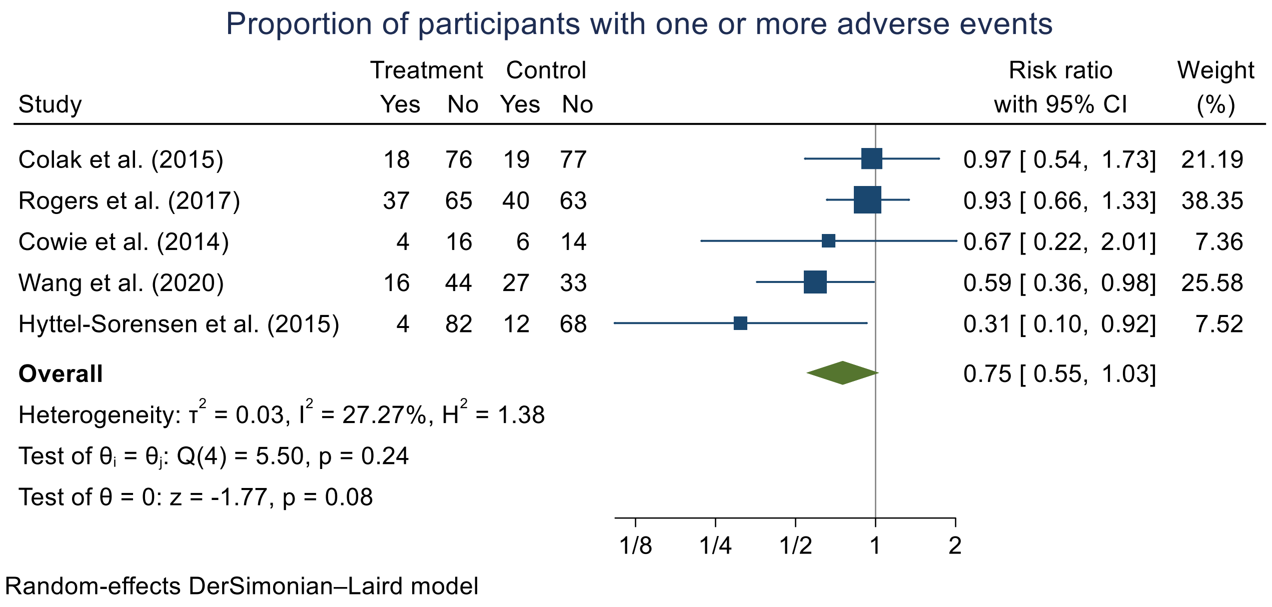

Any evidence of a negative impact on the brain

Primary analysis

Figure S38 – fixed effect

Any evidence of a negative impact on the brain

Study	Treatment Yes	Treatment No	Control Yes	Control No	Risk ratio with 95% CI	Weight (%)
Colak et al. (2015)	28	60	52	41	0.57 [0.40, 0.81]	15.54
Deschamps et al. (2016)	4	30	7	39	0.77 [0.25, 2.43]	1.83
Girgin et al. (2015)	6	44	10	40	0.60 [0.24, 1.53]	3.07
Kara et al. (2015)	7	36	19	17	0.31 [0.15, 0.65]	6.36
Lei et al. (2017)	30	94	31	95	0.98 [0.64, 1.52]	9.45
Mohandas et al. (2013)	2	48	34	16	0.06 [0.01, 0.23]	10.45
Murkin et al. (2007)	1	99	4	96	0.25 [0.03, 2.20]	1.23
Sahan et al. (2018)	7	12	10	11	0.77 [0.37, 1.62]	2.92
Rogers et al. (2017)	2	97	1	105	2.14 [0.20, 23.25]	0.30
Casati et al. (2005)	20	36	30	36	0.79 [0.51, 1.22]	8.46
Cowie et al. (2014)	0	20	0	20	1.00 [0.02, 48.09]	0.15
Murniece et al. (2019)	3	20	4	7	0.36 [0.10, 1.33]	1.66
Wang et al. (2020)	11	49	22	38	0.50 [0.27, 0.94]	6.76
Zogogiannis et al. (2011)	21	146	11	75	0.98 [0.50, 1.94]	4.46
Hyttel-Sorensen et al. (2015)	59	21	51	26	1.11 [0.91, 1.37]	15.97
Pichler et al. (2016)	4	11	6	9	0.67 [0.23, 1.89]	1.84
Leal-Noval et al. (2017)	26	25	31	20	0.84 [0.59, 1.19]	9.53
**Overall**					0.69 [0.61, 0.79]	

Heterogeneity: I^2 = 66.18%, H^2 = 2.96

Test of $\theta_i = \theta_j$: Q(16) = 47.31, p = 0.00

Test of θ = 0: z = -5.39, p = 0.00

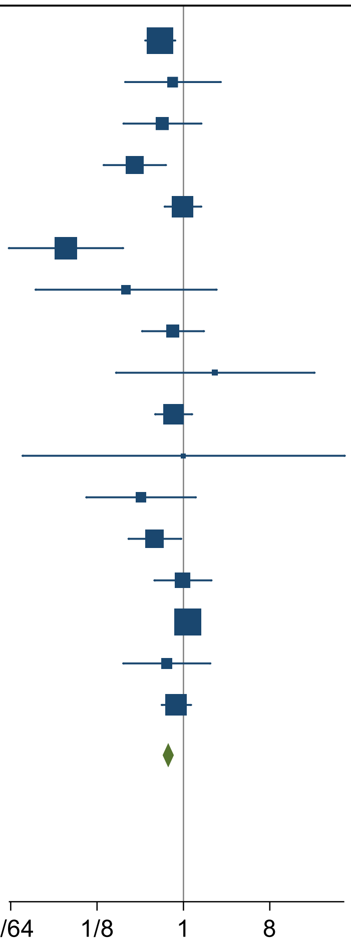

Fixed-effects Mantel–Haenszel model

Figure S39 – random effects

Study	Treatment Yes	Treatment No	Control Yes	Control No	Risk ratio with 95% CI	Weight (%)
Colak et al. (2015)	28	60	52	41	0.57 [0.40, 0.81]	10.57
Deschamps et al. (2016)	4	30	7	39	0.77 [0.25, 2.43]	3.59
Girgin et al. (2015)	6	44	10	40	0.60 [0.24, 1.53]	4.77
Kara et al. (2015)	7	36	19	17	0.31 [0.15, 0.65]	6.21
Lei et al. (2017)	30	94	31	95	0.98 [0.64, 1.52]	9.56
Mohandas et al. (2013)	2	48	34	16	0.06 [0.01, 0.23]	2.73
Murkin et al. (2007)	1	99	4	96	0.25 [0.03, 2.20]	1.24
Sahan et al. (2018)	7	12	10	11	0.77 [0.37, 1.62]	6.26
Rogers et al. (2017)	2	97	1	105	2.14 [0.20, 23.25]	1.05
Casati et al. (2005)	20	36	30	36	0.79 [0.51, 1.22]	9.52
Cowie et al. (2014)	0	20	0	20	1.00 [0.02, 48.09]	0.42
Murniece et al. (2019)	3	20	4	7	0.36 [0.10, 1.33]	2.92
Wang et al. (2020)	11	49	22	38	0.50 [0.27, 0.94]	7.33
Zogogiannis et al. (2011)	21	146	11	75	0.98 [0.50, 1.94]	6.80
Hyttel-Sorensen et al. (2015)	59	21	51	26	1.11 [0.91, 1.37]	12.25
Pichler et al. (2016)	4	11	6	9	0.67 [0.23, 1.89]	4.11
Leal-Noval et al. (2017)	26	25	31	20	0.84 [0.59, 1.19]	10.66
**Overall**					0.67 [0.52, 0.87]	

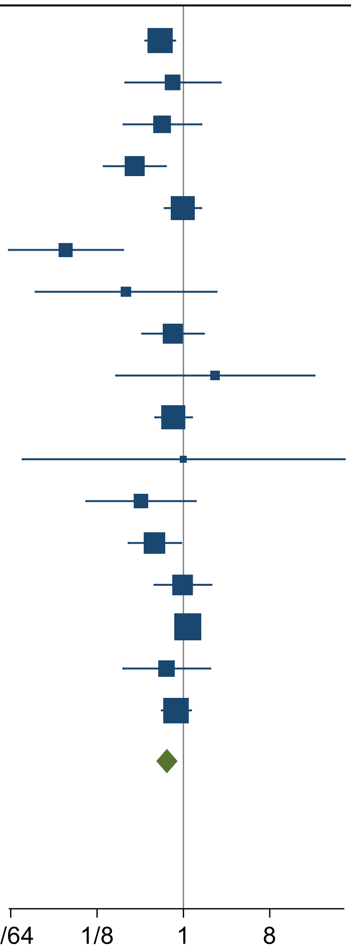

Heterogeneity: $\tau^2 = 0.13$, $I^2 = 60.60\%$, $H^2 = 2.54$

Test of $\theta_i = \theta_j$: Q(16) = 40.61, p = 0.00

Test of $\theta = 0$: z = -3.07, p = 0.00

Random-effects DerSimonian–Laird model

Sensitivity analysis

Heterogeneity (excluding Mohandas et al. 2013)

Figure S40

Any evidence of a negative impact on the brain (excluding Mohandas et al 2013)

Study	Treatment Yes	Treatment No	Control Yes	Control No	Risk ratio with 95% CI	Weight (%)
Colak et al. (2015)	28	60	52	41	0.57 [0.40, 0.81]	12.28
Deschamps et al. (2016)	4	30	7	39	0.77 [0.25, 2.43]	2.81
Girgin et al. (2015)	6	44	10	40	0.60 [0.24, 1.53]	3.94
Kara et al. (2015)	7	36	19	17	0.31 [0.15, 0.65]	5.53
Lei et al. (2017)	30	94	31	95	0.98 [0.64, 1.52]	10.38
Murkin et al. (2007)	1	99	4	96	0.25 [0.03, 2.20]	0.87
Sahan et al. (2018)	7	12	10	11	0.77 [0.37, 1.62]	5.58
Rogers et al. (2017)	2	97	1	105	2.14 [0.20, 23.25]	0.73
Casati et al. (2005)	20	36	30	36	0.79 [0.51, 1.22]	10.31
Cowie et al. (2014)	0	20	0	20	1.00 [0.02, 48.09]	0.28
Murniece et al. (2019)	3	20	4	7	0.36 [0.10, 1.33]	2.21
Wang et al. (2020)	11	49	22	38	0.50 [0.27, 0.94]	6.95
Zogogiannis et al. (2011)	21	146	11	75	0.98 [0.50, 1.94]	6.26
Hyttel-Sorensen et al. (2015)	59	21	51	26	1.11 [0.91, 1.37]	16.11
Pichler et al. (2016)	4	11	6	9	0.67 [0.23, 1.89]	3.29
Leal-Noval et al. (2017)	26	25	31	20	0.84 [0.59, 1.19]	12.46
**Overall**					0.74 [0.60, 0.91]	

Heterogeneity: $\tau^2 = 0.06$, $I^2 = 42.99\%$, $H^2 = 1.75$

Test of $\theta_i = \theta_j$: $Q(15) = 26.31$, $p = 0.03$

Test of $\theta = 0$: $z = -2.82$, $p = 0.00$

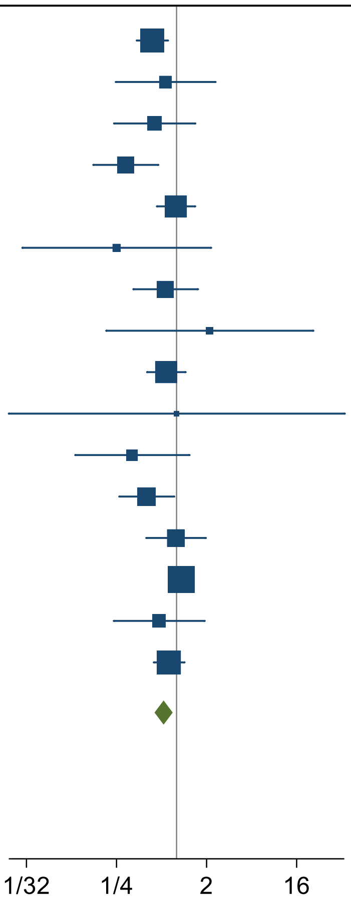

Random-effects DerSimonian–Laird model

Appendix E: Datafile for statistical analysis program

Please go to

LINK FIGSHARE: https://figshare.com/s/2b667e81c89b031f2156

For full access to the datafile used in Stata to conduct the statistical analyses for this systematic review with meta-analysis

Appendix F: Synthax for STATA/SE 17.0

Please go to

LINK FIGSHARE: https://figshare.com/s/dd48f8aec10b1718e83f

For full access to the Synthax used in Stata to conduct the statistical analyses for this systematic review with meta-analysis

Appendix G: Correspondence with the trialists

Please go to

LINK FIGSHARE: https://figshare.com/s/9bf7f10d3e82ca7924d4

For full access to the correspondence with the trialists in order to obtain unpublished data.
